# Task-Dependent Recruitment of Modality-Specific and Multimodal Regions during Conceptual Processing

**DOI:** 10.1093/cercor/bhaa010

**Published:** 2020-03-06

**Authors:** Philipp Kuhnke, Markus Kiefer, Gesa Hartwigsen

**Affiliations:** 1 Lise Meitner Research Group ‘Cognition and Plasticity’, Max Planck Institute for Human Cognitive and Brain Sciences, Stephanstr. 1a, 04103 Leipzig, Germany; 2 Department of Neuropsychology, Research Group ‘Modulation of Language Networks’, Max Planck Institute for Human Cognitive and Brain Sciences, Stephanstr. 1a, 04103 Leipzig, Germany; 3 Department of Psychiatry, Ulm University, Leimgrubenweg 12, 89075 Ulm, Germany

**Keywords:** concepts, embodied cognition, fMRI, language, semantic memory

## Abstract

Conceptual knowledge is central to cognitive abilities such as word comprehension. Previous neuroimaging evidence indicates that concepts are at least partly composed of perceptual and motor features that are represented in the same modality-specific brain regions involved in actual perception and action. However, it is unclear to what extent the retrieval of perceptual–motor features and the resulting engagement of modality-specific regions depend on the concurrent task. To address this issue, we measured brain activity in 40 young and healthy participants using functional magnetic resonance imaging, while they performed three different tasks—lexical decision, sound judgment, and action judgment—on words that independently varied in their association with sounds and actions. We found neural activation for sound and action features of concepts selectively when they were task-relevant in brain regions also activated during auditory and motor tasks, respectively, as well as in higher-level, multimodal regions which were recruited during both sound and action feature retrieval. For the first time, we show that not only modality-specific perceptual–motor areas but also multimodal regions are engaged in conceptual processing in a flexible, task-dependent fashion, responding selectively to task-relevant conceptual features.

## Introduction

Conceptual knowledge about objects, people, and events in the world is crucial for many cognitive abilities such as recognizing and acting with objects and understanding the meaning of words (Barsalou 1999; Binder and Desai 2011; Kiefer and Pulvermüller 2012; Lambon Ralph 2014). Thus, a central question in cognitive neuroscience has been how concepts are represented and processed in the human brain.

Grounded theories of conceptual representation propose that concepts consist of perceptual and motor features, which are represented in the same modality-specific brain systems engaged during actual perception and action (Barsalou 2008; Kiefer and Pulvermüller 2012; Hauk and Tschentscher 2013; Kiefer and Barsalou 2013). For instance, sound features of concepts are represented in auditory brain regions, while action features are represented in motor-related regions. Evidence for grounded theories is provided by neuroimaging studies demonstrating that processing concepts related to a certain perceptual–motor modality activates the respective modality-specific brain regions (for reviews, see Binder and Desai 2011; Kiefer and Pulvermüller 2012; Hauk and Tschentscher 2013; Borghesani and Piazza 2017). For example, processing action verbs engages the motor cortex (Hauk et al. 2004; Hauk and Pulvermüller 2004), while processing sound-related words activates auditory association regions, such as posterior middle temporal gyrus (pMTG; Kiefer et al. 2008, 2012b; Fernandino et al. 2016a). Moreover, lesions of motor or auditory brain regions are associated with deficits in action-related (Bak et al. 2001, 2006; Hillis et al. 2006; Grossman et al. 2008) or sound-related conceptual processing (Bonner and Grossman 2012; Trumpp et al. 2013a), respectively.

In addition to modality-specific areas, previous evidence suggests that conceptual processing involves “convergence zones” (Damasio 1989) at multiple hierarchical levels, which integrate modality-specific representations into increasingly abstract representations (Simmons and Barsalou 2003; Binder et al. 2009; Binder 2016). Therefore, several researchers propose conceptual processing to rely on a hierarchy of functional neural networks from modality-specific to multimodal (i.e., bimodal, trimodal, etc.) up to heteromodal areas (Simmons and Barsalou 2003; Binder and Desai 2011; Kiefer and Pulvermüller 2012; Garagnani and Pulvermüller 2016). Although a common terminology is currently lacking in the field, we call regions “modality-specific” if they represent information related to a single perceptual–motor modality and are located within perceptual–motor systems (following grounded views). We refer to areas as “multimodal” if they integrate information from at least two modalities and remain sensitive to the individual modalities. “Heteromodal” regions are areas that receive input from all modalities. A heteromodal region can be either multimodal itself (i.e., sensitive to individual perceptual-motor modalities) or “amodal” (i.e., insensitive to individual modalities). “Cross-modal” is an overarching term for any region that integrates at least two modalities and thus subsumes multimodal and heteromodal areas. Previous evidence indicates that high-level cross-modal convergence zones are located in the posterior inferior parietal lobe (pIPL), pMTG, medial prefrontal cortex (mPFC) (Binder et al. 2009; Binder 2016), and anterior temporal lobe (ATL; Lambon Ralph et al. 2016).

However, it is unclear to what extent the retrieval of perceptual–motor features and the involvement of modality-specific regions in conceptual processing depend on the concurrent task. According to one view, perceptual–motor features are always activated in a task-independent fashion (Pulvermüller 2005). This view is supported by studies demonstrating activation of modality-specific areas during implicit conceptual tasks (e.g., lexical decision; Pulvermüller et al. 2005; Kiefer et al. 2008, 2012a) or even passive tasks (e.g., Hauk et al. 2004; Hauk and Pulvermüller 2004). Such modality-specific recruitment can occur as early as 200 ms after stimulus onset (Hauk and Pulvermüller 2004; Kiefer et al. 2008), and even when stimuli are unattended (Shtyrov et al. 2004; Pulvermüller and Shtyrov 2006) or not consciously perceived (Trumpp et al. 2013b, 2014).

In contrast, other studies suggest that the retrieval of perceptual–motor features varies with the task. Behavioral studies indicate that even central features of a concept, including perceptual–motor features, can be modulated by the context such as the task (for reviews, see Kiefer et al. 2012b; Lebois et al. 2015). Moreover, evidence from neuroimaging (Bedny et al. 2008; Postle et al. 2008; Raposo et al. 2009), transcranial magnetic stimulation (Papeo et al. 2009, 2015), and lesion studies (Arévalo et al. 2012; Kemmerer et al. 2012) suggests that activation of modality-specific areas does not always occur during conceptual processing.

Some authors have taken the absence of modality-specific activity during some tasks as evidence against grounded theories (e.g., Bedny et al. 2008; Papeo et al. 2009; Mahon 2015). In contrast, proponents of grounded theories have argued that such variability could be meaningful and systematic, reflecting the fact that the retrieval of perceptual–motor features and corresponding recruitment of modality-specific brain regions occurs flexibly in a task-dependent fashion (Hoenig et al. 2008; Kemmerer 2015; Barsalou 2016; Pulvermüller 2018). Specifically, depending on the task explicitness and relevance of perceptual–motor features, different levels of the processing hierarchy may be recruited: An implicit task that does not require perceptual–motor information might only involve high-level convergence zones, whereas a task that explicitly requires retrieval of perceptual–motor features may additionally recruit lower-level perceptual–motor areas (Kemmerer 2015; Popp et al. 2019a). For instance, Binder and colleagues propose that high-level cross-modal areas (e.g., pIPL, pMTG, mPFC, and ATL) are consistently engaged in conceptual processing in a task-independent fashion, whereas the recruitment of modality-specific perceptual–motor areas is assumed to depend on contextual factors such as the task (Binder and Desai 2011; Fernandino et al. 2016a). Tackling the issue of task dependency is therefore crucial to refine theories of conceptual processing and specify how different levels of the processing hierarchy are engaged under different circumstances (Binder and Desai 2011; Willems and Casasanto 2011; Yee and Thompson-Schill 2016).

Although very few neuroimaging studies have directly tested the task dependency of conceptual processing so far, these studies generally support the view that the retrieval of perceptual–motor features and the engagement of modality-specific brain regions depend on the task. For example, Hoenig et al. (2008) found that visual- and motor-related areas showed stronger activity when a nondominant feature had to be verified for a concept. Another study reported several motor-related regions to be more active for words with a high relevance of both action and color features when the task focused on action as opposed to color (van Dam et al. 2012). Hsu et al. (2011) showed that a task which required more detailed color knowledge engaged color-sensitive cortex to a stronger degree. Finally, Borghesani et al. (2019) found areas associated with motion and action processing to exhibit higher activity when two objects are compared for movement than for typical location.

However, these studies have several limitations. Firstly, they exclusively focused on visual and action features, whereas little is known about other modalities such as sound. Moreover, except for Hsu et al., none of the previous studies tested for activation overlap with actual perception and action. Consequently, it remains unknown whether the activated regions were indeed located within perceptual–motor systems. In addition, Hsu et al. and Hoenig et al. confounded their task manipulation with stimulus manipulations, rendering it unclear whether activation differences were due to different tasks, different stimuli, or both. Finally, no previous study independently manipulated the relevance of multiple perceptual–motor features at the same time. It thus remains unknown whether the implicated regions were indeed modality-specific or rather multimodal.

To address these issues and systematically investigate to what extent neural activity for perceptual–motor features of concepts depends on the task, the present functional magnetic resonance imaging (fMRI) study compared different tasks on the same stimuli in the same participants and directly tested for activation overlap with perception and action. Participants performed three different tasks on words that exhibited either a low or high association with sounds and actions, thereby orthogonally varying task and feature relevance. A lexical decision task probed implicit access to action and sound features of concepts, whereas action and sound judgment tasks assessed explicit retrieval of action and sound features, respectively.

Following grounded theories, we hypothesized that retrieval of action features should engage motor-related brain regions, while retrieval of sound features should engage auditory-related regions. Moreover, based on previous work (e.g., Binder and Desai 2011; Fernandino et al. 2016a), we expected that feature-related activity in modality-specific perceptual–motor regions should be increased when the respective feature is task-relevant, whereas activity of high-level cross-modal regions should not be modulated by task.

We found activation for sound or action features exclusively when they were task-relevant. In line with grounded theories, activation for sound or action features overlapped with sound perception or motor action, respectively. However, activation extended beyond auditory or motor areas to higher-level, multimodal regions, which were engaged for both sound and action features. As an unexpected, novel finding, not only modality-specific areas but also multimodal regions showed a flexible, task-dependent recruitment pattern, responding selectively to task-relevant conceptual features. These findings indicate that the task modulates not only which levels of the processing hierarchy (modality-specific, multimodal, up to heteromodal regions) are engaged. The task also influences the neural response to individual perceptual–motor features of concepts at several hierarchy levels, even including high-level cross-modal convergence zones.

## Materials and Methods

### Subjects

Data from 40 native German speakers (22 females; mean age: 26.6 years; SD: 4.1; range: 19–33) entered the final analysis. A total of 42 subjects were initially recruited, but 2 were excluded due to stopping the experiment or excessive head movement. All subjects were right-handed (mean laterality quotient: 93.7; SD: 9.44; Oldfield 1971). No subject had a history of neurological disorders or head injury or exhibited contraindications to fMRI. All subjects were recruited via the subject database of the Max Planck Institute for Human Cognitive and Brain Sciences, Leipzig, Germany. Written informed consent was obtained from each subject prior to the experiment. The study was performed according to the guidelines of the Declaration of Helsinki and approved by the local ethics committee of the University of Leipzig.

### Experimental Procedures

In two event-related fMRI sessions on separate days, subjects performed three different tasks on the same 192 words that independently varied in their association strength with sounds and actions. The experiment thus followed a 3 × 2 × 2 repeated-measures design with the factors TASK (lexical decision, sound judgment, action judgment), SOUND (low, high relevance for word meaning), and ACTION (low, high relevance for word meaning). All stimuli were presented using the software *Presentation* (Neurobehavioral Systems, Inc., Berkeley, CA, www.neurobs.com; version 17.2). Visual stimuli were back-projected onto a mirror mounted on the head coil. Auditory stimuli were played via MR-compatible in-ear headphones (MR Confon, Magdeburg, Germany).

#### Session 1. Lexical Decision Task (Implicit)

In the first session, subjects performed a lexical decision task. On each trial, they decided whether the presented stimulus was a word or pseudoword. This implicit conceptual task did not require explicit retrieval of sound or action features. The lexical decision task was always performed before the explicit tasks (see below) to ensure that the subjects’ attention was not directed toward the sound or action features of the words.

**Figure 1:**
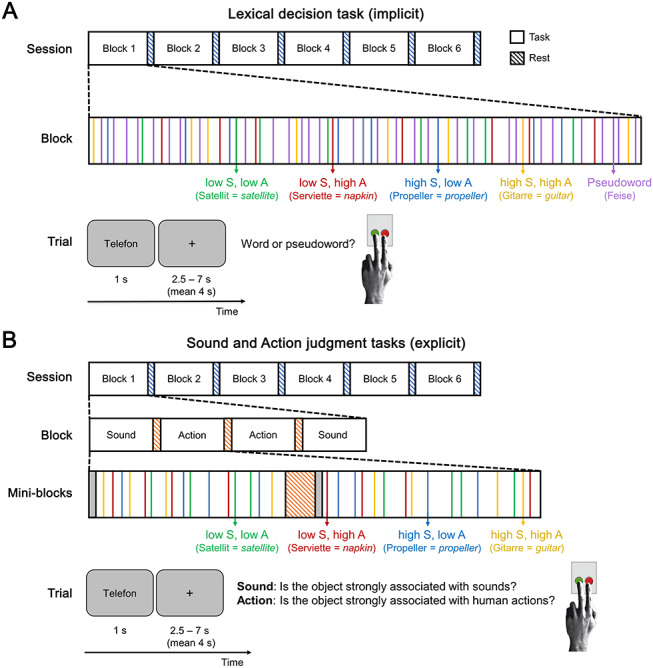
Experimental design. An experimental session consisted of six blocks separated by 20-s rest periods (blue-striped bars). In session 1 (*A*), each block contained 64 trials presented in random order: 32 trials of pseudowords (purple) and 32 trials of real words. These included eight trials for each word category: low sound, low action (green); low sound, high action (red); high sound, low action (blue); high sound, high action (yellow). During each trial, a letter string was shown for 1 s and subjects performed a lexical decision, followed by a jittered inter-trial-interval (ITI) of 2.5–7 s (mean 4 s). In session 2 (*B*), each block included four mini-blocks—two of sound judgments and two of action judgments—which were separated by 12-s rest periods (orange-striped bars). Each mini-block started with a cue indicating the task (gray bars; Supplementary Fig. S1). Then, 16 trials followed, with four trials per word category. In each trial, subjects judged whether the presented word was strongly associated with sounds (sound judgment) or whether it was strongly associated with actions (action judgment).

A total of 384 trials (192 words, 192 pseudowords) were presented in six blocks, separated by 20-s fixation period during which subjects could rest (Fig. 1*A*). Subjects responded via button press with the index or middle finger of their left hand. They were instructed to respond as quickly and as accurately as possible. Button assignment was counterbalanced across subjects.

The length of the scanning session was ~35 min. Before entering the scanner, subjects practiced the task with 16 trials that were not included in the actual experiment.

#### Session 2. Sound and Action Judgment Tasks (Explicit)

In the second session, two explicit conceptual tasks were performed. In the sound judgment task, subjects decided whether the presented word was strongly associated with sounds or not. In the action judgment task, subjects judged whether the word was strongly associated with actions. Sound judgments thus explicitly required retrieval of sound features, whereas action judgments required retrieval of action features of concepts. The two tasks were performed in separate mini-blocks to ensure a constant cognitive state during each task and minimize task switching effects.

As in session 1, 384 trials were presented in six blocks of 64 trials each (Fig. 1*B*). Each of the 192 words was presented in both tasks (counterbalanced across subjects). The order of mini-blocks was pseudo-randomized with the restriction that the same condition could not occur more than twice in succession. Trials were presented in a pseudo-randomized order within blocks with the restriction that all words were presented before any word was repeated and that word repetitions were separated by at least two mini-blocks.

The overall length of the scanning session was ~38 min. Subjects practiced both tasks outside the scanner before the session with 16 trials excluded from the main experiment.

### Stimuli

Stimuli were 192 written German nouns denoting concrete objects, which were strongly or weakly associated with sounds and (human) actions, leading to four categories of 48 words each: 1) low sound, low action; 2) low sound, high action; 3) high sound, low action; and 4) high sound, high action (see Fig. 1 for examples).

A total of 163 subjects who did not participate in the fMRI experiment rated an original set of 891 words for their association with sounds, actions, and visual features, as well as their familiarity on a 1-to-6 scale (for a similar procedure, see Kiefer et al. 2008; Bonner et al. 2013; Trumpp et al. 2014; Fernandino et al. 2016b). We selected 48 words for each category such that high and low sound words differed selectively in their sound ratings (*P* < 10^−113^), while high and low action words differed only in their action ratings (*P* < 10^−103^). Categories were matched on all other rating criteria and further psycholinguistic measures, including number of letters and syllables, word frequency, bi- and trigram frequencies, and number of orthographic neighbors (all *P* > 0.05; Supplementary Table S1). Note that stimuli for the four word categories were drawn from the same superordinate categories of animals, inanimate natural entities, and man-made objects (cf. Goldberg et al. 2006; Kiefer et al. 2008).

For the lexical decision task, 192 phonologically and orthographically legal pseudowords were created using the software *Wuggy* (Keuleers and Brysbaert 2010; http://crr.ugent.be/Wuggy). For each real word in the experiment, a pseudoword was generated that was matched in length, syllable structure, and transition frequencies between subsyllabic elements.

### Functional Localizers

At the end of the second session, two functional localizers were administered to determine auditory and motor brain regions, respectively. Their order was counterbalanced across subjects.

In the auditory localizer, participants were presented with blocks of 1) real object sounds and 2) scrambled versions of the same sounds. Real object sounds should engage high-level auditory representations (e.g., barking of a dog; Bizley and Cohen 2013), whereas scrambled sounds should exclusively recruit low-level acoustic representations (e.g., frequency, loudness). Subjects were instructed to attentively listen to the sounds, while maintaining fixation on a cross (cf. Kiefer et al. 2008; Hoenig et al. 2011). Sounds were presented in 12 blocks (6 real, 6 scrambled) of 18 s each and interspersed with 16-s silence blocks. Block order alternated between real and scrambled sounds. Real sounds included sounds of animals (e.g., elephant), inanimate natural entities (e.g., river), tools (e.g., saw), musical instruments (e.g., violin), and everyday objects (e.g., telephone). Scrambled sounds were created in *Matlab* (version 9.3/2017b) as described by Dormal et al. (2018), yielding sounds that were well-matched to the real sounds for low-level acoustic features but did not have any meaning. All sounds were matched for root mean square intensity, and a 5-ms fade was added at the beginning and end of each sound to avoid click artifacts (Belin et al. 2000; Dormal et al. 2018). The length of the auditory localizer was ~8 min.

In the motor localizer, participants performed three types of movements with their left or right hand in separate blocks, including finger tapping (sequence from thumb to little finger), fist making, and pinching (cf. Bonner et al. 2013). A written cue indicated the type of movement and hand at the beginning of each block. Movement was paced by a fixation cross blinking in a 1 Hz rhythm. Subjects performed 12 movement blocks (2 per movement type per hand) of 18 s, separated by 16-s rest blocks during which the same visual stimulus (blinking cross) was shown but no movements were executed. The motor localizer took ~9 min.

### fMRI Data Acquisition and Preprocessing

FMRI data were collected on a 3T Prisma scanner (Siemens, Erlangen, Germany) with a 32-channel head coil. Functional, blood oxygenation level-dependent (BOLD) images were acquired using a multiband (Feinberg et al. 2010) dual gradient-echo EPI sequence (repetition time [TR]: 2 s; echo time [TE]: 12 & 33 ms; flip angle: 90°; field of view [FoV]: 204 mm; voxel size: 2.5 × 2.5 × 2.5 mm; slice gap: 0.25 mm; bandwidth: 1966 Hz/Px; phase encoding direction: A/P; acceleration factor 2). We used a dual-echo sequence to maximize BOLD sensitivity throughout the whole brain, including regions susceptible to signal loss in standard single-echo EPI, such as the ATL (Poser et al. 2006; Halai et al. 2014). To further reduce susceptibility artifacts, slices were tilted 10° up (at anterior edge) from the AC-PC line (Weiskopf et al. 2006). Sixty slices covering the whole brain were recorded in interleaved order and axial orientation. B0 field maps were acquired for susceptibility distortion correction using a gradient-echo sequence (TR: 0.62 s; TE: 4 & 6.46 ms; flip angle: 60°; bandwidth: 412 Hz/Px; other parameters identical to functional sequence). Structural T1-weighted images were acquired for normalization using an MPRAGE sequence (176 slices in sagittal orientation; TR: 2.3 s; TE: 2.98 ms; FoV: 256 mm; voxel size: 1 × 1 × 1 mm; no slice gap; flip angle: 9°; phase encoding direction: A/P).

fMRI analysis was performed using *Statistical Parametric Mapping* (SPM12; Wellcome Trust Centre for Neuroimaging; http://www.fil.ion.ucl.ac.uk/spm/), implemented in *Matlab* (version 9.3/2017b). The two images with a short and long TE were combined using an average weighted by the temporal signal-to-noise ratio (tSNR) of each image at every voxel, which yields optimal BOLD sensitivity at each voxel (Poser et al. 2006). tSNR was calculated based on 30 volumes collected at the beginning of each scanning run, which were excluded from further analyses. Functional images were realigned, distortion corrected (using a B0 field map), slice-timing corrected, normalized to MNI space via unified segmentation of the co-registered structural image (resampling to 2.5 mm^3^ isotropic voxels), and smoothed with an 8 mm^3^ FWHM Gaussian kernel.

### Whole-Brain Analyses

We performed a whole-brain random-effects group analysis based on the general linear model (GLM), using the two-level approach in SPM. At the first level, individual subject data were modeled separately. For the localizers, blocks were modeled using box-car regressors convolved with the canonical hemodynamic response function (HRF). For the conceptual tasks, the GLM included regressors for the 12 experimental conditions, modeling trials as stick functions convolved with the canonical HRF and its temporal derivative. Only correct trials were analyzed, error trials were modeled in a separate regressor-of-no-interest. To account for potential differences in response time (RT) between trials and conditions, a duration-modulated parametric regressor (duration = RT) was included (Grinband et al. 2008). For all tasks, nuisance regressors included the six motion parameters and individual regressors for time points with strong volume-to-volume movement (framewise displacement > 0.9; Siegel et al. 2014). The data were subjected to an AR(1) autocorrelation model to account for temporal autocorrelations and high-pass filtered (cutoff 128 s) to remove low-frequency noise.

Contrast images for each participant were computed at the first level. At the second level, these contrast images were submitted to one-sample or paired *t*-tests (to test for interactions). To identify brain regions sensitive to action or sound features in each task (lexical decision, action judgment, sound judgment), we first compared activation for high > low action words and high > low sound words within each task. Conjunction analyses based on the minimum statistic (testing the conjunction null hypothesis; Nichols et al. 2005) tested for overlap between activation for action or sound features and activation in the motor localizer (hand movements > rest) or auditory localizer (real object sounds > silence; scrambled sounds > silence), respectively.

To localize brain regions whose response to sound or action features depended on the task, we directly compared the activation for high > low action words and high > low sound words between tasks using paired *t*-tests. We contrasted high > low action or sound words within each task first to isolate task-specific activity for action or sound features, while controlling for other potential differences between tasks (such as condition-unspecific differences in response magnitude). To restrict interactions to voxels significant within the task, interactions were inclusively masked by significant voxels of the minuend (cf. Noppeney et al. 2006; Hardwick et al. 2018). We corrected for multiple comparisons at the whole-brain level using false discovery rate (FDR) correction (see below).

Finally, we aimed to localize regions involved in the explicit retrieval of both sound and action features. To this end, a conjunction analysis was performed between [high > low action words during action judgments] and [high > low sound words during sound judgments].

For all second-level analyses, a gray matter mask was applied, restricting statistical tests to voxels with a gray matter probability > 0.3 (SPM12 tissue probability map). All activation maps were thresholded at a voxel-wise FDR of *q* < 0.05 (Benjamini and Hochberg 1995; Genovese et al. 2002), with an additional cluster extent threshold of 20 voxels.

### Subject-Specific Functional Region of Interest Analysis

To characterize the response profiles of motor, auditory, and multimodal regions identified in individual subjects, we performed a functional region of interest (fROI) analysis (Fedorenko et al. 2010; Nieto-Castañón and Fedorenko 2012) using the group-constrained subject-specific (GSS) approach (Julian et al. 2012).

We defined three types of fROIs: 1) “Motor fROIs”—motor regions involved in action feature retrieval—using the conjunction [Action judgment: high > low action words] ∩ [Motor localizer: hand movements > rest], 2) “Auditory fROIs”—auditory regions involved in sound feature retrieval—using the conjunction [Sound judgment: high > low sound words] ∩ [Auditory localizer: real sounds > silence], and 3) “Multimodal fROIs”—regions involved in both action and sound feature retrieval—using the conjunction [Action judgment: high > low action words] ∩ [Sound judgment: high > low sound words]. Motor and auditory fROIs were defined via overlap with the motor and auditory localizers to identify grounded conceptual regions, whereas multimodal fROIs could be higher-level areas not involved in basic action or perception. To avoid circularity (Kriegeskorte et al. 2009; Vul et al. 2009), we employed a split-half approach, using half of the data of each subject (blocks 1–3) for fROI definition and the other half (blocks 4–6) for response estimation (cf. Fedorenko et al. 2011, 2013).

fROI definition followed the GSS procedure (Julian et al. 2012): For each fROI type, subject-specific activation maps (5-mm smoothing) were thresholded at *P* < 0.05 and overlaid on top of each other; the resulting overlap map showed how many subjects exhibited activation at each voxel. The overlap map was smoothed (5 mm), thresholded at two subjects (cf. Julian et al. 2012), and parcellated using a watershed algorithm (Meyer 1991) implemented in the spm_ss toolbox (Nieto-Castañón and Fedorenko 2012). We retained only those parcels within which at least 60% of subjects had any suprathreshold voxels or for which we had a priori hypotheses (cf. Fedorenko et al. 2010; Julian et al. 2012). To maximize generalizability to the population, the final analysis included all subjects: fROIs were defined in each individual subject as the 10% most active voxels for the conceptual contrast within each parcel (Fedorenko et al. 2012; Basilakos et al. 2018). Finally, using exclusively the left-out data, percent signal change was estimated for each fROI and condition using the MarsBaR toolbox (Brett et al. 2002).

Statistical inference was performed using a four-way repeated-measures ANOVA (Greenhouse-Geisser corrected) with the factors REGION (all fROIs), TASK (lexical decision, sound judgment, action judgment), SOUND (high, low), and ACTION (high, low). Interactions were resolved using step-down analyses and Bonferroni-Holm corrected post hoc comparisons. We report results for fROIs with significant effects.

## Results

### Behavioral Results

Mean response time for correct responses was 971.62 ms (SD: 157.10 ms). Mean accuracy was 92.28% (SD: 4.43%), which shows that subjects closely attended to the tasks.

**Figure 2:**
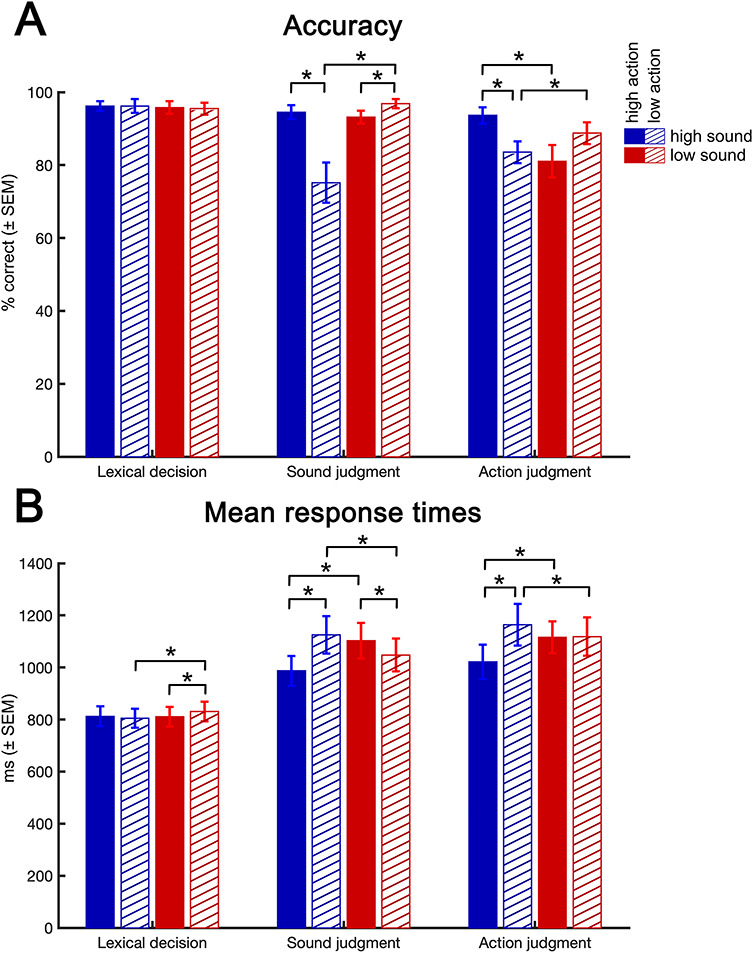
Behavioral results. (*A*) Accuracy is shown in percent correct responses. (*B*) Mean response times for correct trials are given in ms. Error bars represent standard error of the mean (SEM). ^*^P < 0.05 (Bonferroni-Holm corrected).

For response accuracy (Fig. 2*A*), a three-way repeated-measures ANOVA (Greenhouse-Geisser corrected) with factors TASK (lexical decision, sound judgment, action judgment), SOUND (high, low), and ACTION (high, low) identified main effects of TASK (*F*(2,78) = 58.11, *P* < 0.001), SOUND (*F*(1,39) = 10.78, *P* = 0.002), and ACTION (*F*(1,39) = 16.11, *P* < 0.001), as well as interactions between SOUND × ACTION (*F*(1,39) = 112.27, *P* < 0.001), TASK × ACTION (*F*(2,78) = 8.43, *P* = 0.003), TASK × SOUND (*F*(2,78) = 40.55, *P* < 0.001), and TASK × SOUND × ACTION (*F*(2,78) = 35.05, *P* < 0.001). Step-down analyses revealed no significant effects during lexical decisions, whereas SOUND × ACTION interactions occurred during sound judgments (*F*(1,39) = 93.49, *P* < 0.001) and action judgments (*F*(1,39) = 47.48, *P* < 0.001). During sound judgments, we found higher accuracy for high sound words with a high than low action association (*t*(39) = 9.26, *P* < 0.001) and the opposite pattern for low sound words (*t*(39) = 4.7, *P* < 0.001). In addition, a main effect of SOUND (*F*(1,39) = 34.52, *P* < 0.001) reflected higher accuracy for low than high sound words and a main effect of ACTION (*F*(1,39) = 57.01, *P* < 0.001) reflected higher accuracy for high than low action words. During action judgments, high action words with a high versus low sound association were more accurate (*t*(39) = 4.0, *P* < 0.001) and the opposite pattern for low action words (*t*(39) = 7.58, *P* < 0.001). Moreover, a main effect of SOUND (*F*(1,39) = 24.40, *P* < 0.001) indicated higher accuracy for high than low sound words.

Response times for correct trials (Fig. 2*B*) also showed main effects of TASK (*F*(2,78) = 148.434, *P* < 0.001), SOUND (*F*(1,39) = 6.550, *P* = 0.014), and ACTION (*F*(1,39) = 26.038, *P* < 0.001), as well as interactions between SOUND × ACTION (*F*(1,39) = 169.427, *P* < 0.001), TASK × ACTION (*F*(2,78) = 7.761, *P* = 0.005), and TASK × SOUND × ACTION (*F*(2,78) = 71.26, *P* < 0.001). All three tasks exhibited a SOUND × ACTION interaction (lexical decision: *F*(1,39) = 18.02, *P* < 0.001; sound judgment: *F*(1,39) = 139.65, *P* < 0.001; action judgment: *F*(1,39) = 79.33, *P* < 0.001). During lexical decisions, we observed faster responses for low action words with a high than low sound association (*t*(39) = 5.72, *P* < 0.001) and for low sound words with a high than low action association (*t*(39) = 4.11, *P* < 0.001). In addition, a main effect of SOUND (*F*(1,39) = 12.76, *P* < 0.001) indicated faster responses for high than low sound words. During sound judgments, high sound words with a high versus low action association lead to faster responses (*t*(39) = 11.51, *P* < 0.001) and vice versa for low sound words (*t*(39) = 5.58, *P* < 0.001). Moreover, a main effect of ACTION (*F*(1,39) = 31.794, *P* < 0.001) reflected shorter responses for high than low action words. During action judgments, low action words with a low versus high sound association were faster (*t*(39) = 3.98, *P* < 0.001) and vice versa for high action words (*t*(39) = 6.69, *P* < 0.001). A main effect of ACTION (*F*(1,39) = 13.07, *P* < 0.001) indicated faster response times for high than low action words and a main effect of SOUND (*F*(1,39) = 5.797, *P* = 0.021) indicated faster responses for high than low sound words.

These behavioral results illustrate the interaction between the relevance of a certain perceptual–motor feature for a concept and the concurrent task, supporting the notion that the retrieval of perceptual–motor features is task-dependent. To account for potential influences of differences in accuracy or reaction times on brain activation, only correct trials were analyzed, and response times were entered into the subject-level GLM as a duration-modulated parametric regressor (Grinband et al. 2008).

### Localizer Activations

The motor localizer (hand movements > rest) engaged bilateral primary, pre-, and supplementary motor cortices, somatosensory cortices, anterior supramarginal gyrus (aSMG) extending into inferior parietal sulcus (IPS), cerebellum, as well as the lateral temporal–occipital junction (LTO) at the border of pMTG to anterior occipital cortex (Supplementary Table S2).

In the auditory localizer, scrambled sounds (> silence) activated bilateral early auditory cortex, brainstem, cerebellum, and right inferior frontal gyrus (IFG). Real sounds (>silence) engaged a broader region of bilateral auditory cortex extending into the superior and middle temporal gyri (STG/MTG), as well as dorsomedial prefrontal cortex (dmPFC), left middle frontal gyrus (MFG), IFG, IPS, and middle cingulate cortex (MCC) (Supplementary Table S3).

### Within-Task Activations for Sound and Action Features of Concepts

We first tested for activation increases for sound features (high > low sound words) and action features of concepts (high > low action words) within each task.

#### Action Features

In the lexical decision task, high as compared to low action words did not elicit significant activation in any voxel (at *q* < 0.05 FDR-corrected). Even when reducing the statistical threshold to *P* < 0.001 uncorrected, only the left ventromedial prefrontal cortex (vmPFC)—a high-level, heteromodal region (Binder et al. 2009)—showed activity, whereas no motor-related regions were engaged.

Similarly, in the sound judgment task, we observed no significant activation for action-related words (at *q* < 0.05 FDR-corrected). At *P* < 0.001 uncorrected, the left angular gyrus (AG), SMG, precuneus, and right vmPFC were activated. Most of these regions (AG, precuneus, vmPFC) represent heteromodal regions involved in conceptual processing (Binder et al. 2009; Binder 2016).

**Figure 3:**
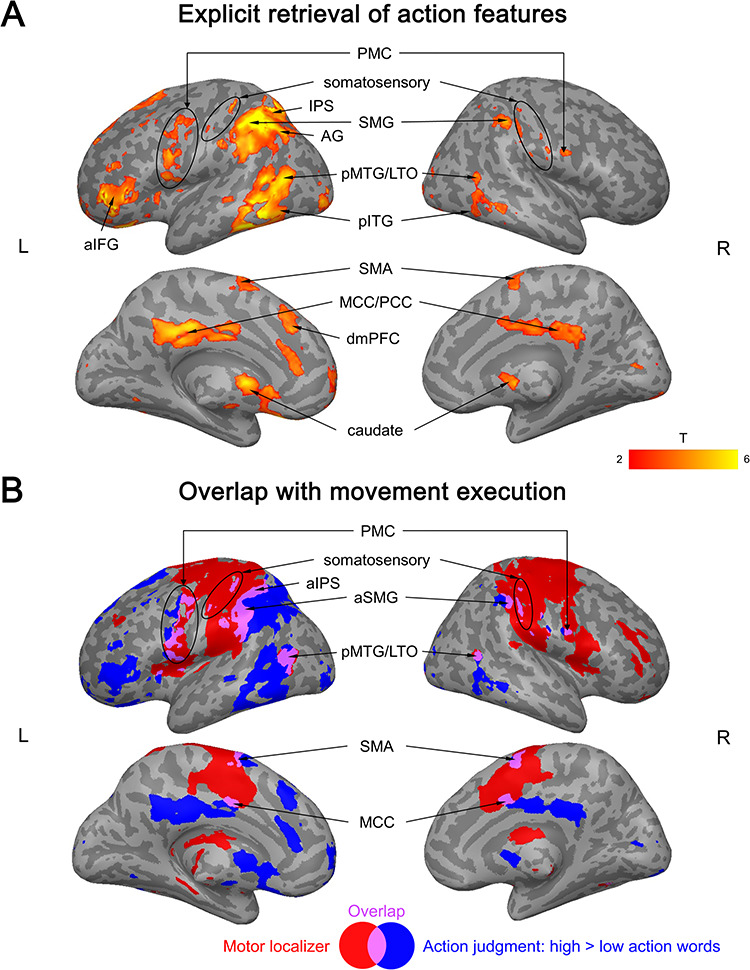
(*A*) Activation for action features (high > low action words) in the action judgment task. (*B*) Overlap (purple) between the activation for action features in the action judgment task (blue) and the motor localizer (hand movements > rest; red). All activation maps were thresholded at *q* < 0.05 FDR-corrected (extent > 20 voxels).

In contrast, in the action judgment task, action-related words produced widespread activation in both hemispheres (Fig. 3*A*; Supplementary Table S4). This activation overlapped with brain activity in the motor localizer in bilateral cerebellum, premotor cortex (PMC), aSMG/IPS, somatosensory cortex, supplementary motor area (SMA), MCC, and pMTG/LTO (Fig. 3*B*; Supplementary Table S5). However, activation for action-related conceptual processing was also present *outside* regions activated by the motor localizer (as determined by exclusive masking), namely in bilateral posterior cingulate cortex (PCC), posterior inferior temporal gyrus (pITG; extending into posterior fusiform gyrus (FG) in the left hemisphere), more posterior parts of SMG/IPS (extending into the superior parietal lobe [SPL] in the left hemisphere), left dmPFC, anterior IFG (aIFG), AG, and more anterior parts of MTG.

#### Sound Features

An analogous pattern emerged for sound features of concepts. In the lexical decision task, no significant activation was found for high versus low sound words, even when reducing the statistical threshold to *P* < 0.001 uncorrected.

Also in the action judgment task, no voxel was significantly activated (at *q* < 0.05 FDR-corrected). An exploratory analysis at *P* < 0.001 uncorrected (extent > 20 voxels) revealed activation in bilateral precuneus/PCC and left posterior IPS.

**Figure 4:**
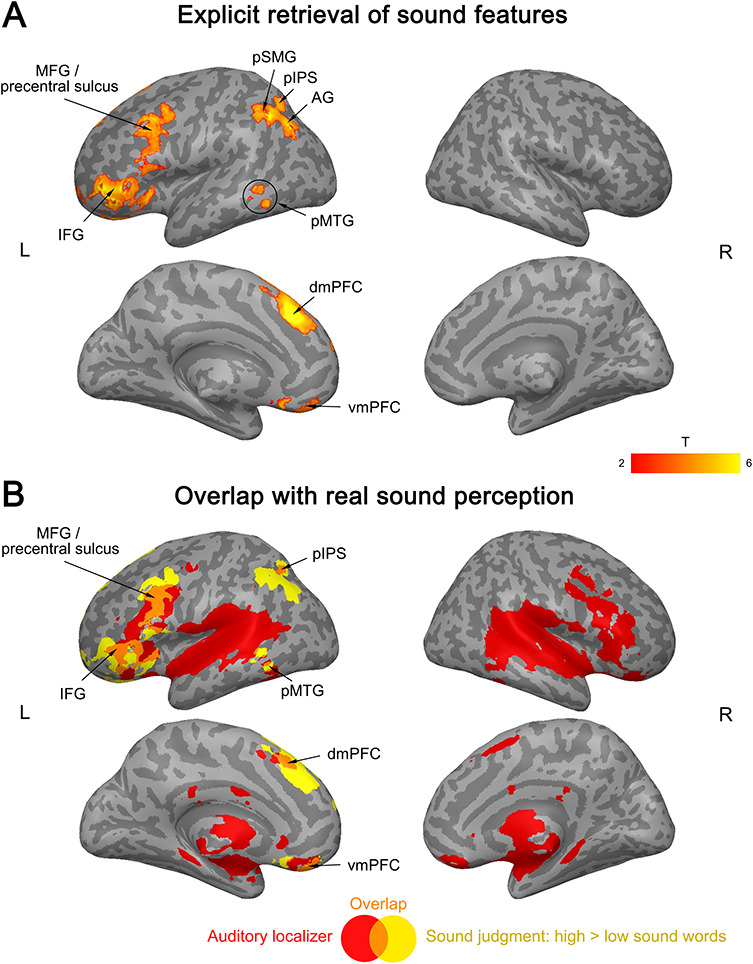
(*A*) Activation for sound features of concepts (high > low sound words) in the sound judgment task. (*B*) Overlap (orange) between activation for sound features in the sound judgment task (yellow) and the auditory localizer (real object sounds > silence; red). All activation maps were thresholded at *q* < 0.05 FDR-corrected (extent > 20 voxels).

In the sound judgment task, however, sound-related words elicited widespread activation (Fig. 4*A*; Supplementary Table S6). This activation did not overlap with brain activity during the perception of scrambled sounds (i.e., sounds that lacked any meaning and mainly engaged early auditory cortices; Supplementary Fig. S3). In contrast, activation for sound-related words during sound judgments overlapped with activity for the perception of real object sounds in left IFG (extending into insula), MFG/precentral sulcus (PreCS), pIPS, pMTG, dmPFC, vmPFC, and right cerebellum (Fig. 4*B*; Supplementary Table S7). However, sound-related words also engaged regions that were not activated during real sound perception, including left AG, posterior SMG (pSMG), and other portions of IFG, MFG/PreCS, pMTG, dmPFC, vmPFC, and right cerebellum.

### Task Dependency of Conceptual Feature Activation

The above-described results suggest that neural activity for a certain conceptual feature is strongly task-dependent: We selectively observed activity for a specific feature in a task that explicitly required that feature. To further investigate the task dependency of activation for action or sound features, we performed several whole-brain interaction analyses.

#### Action Features

**Figure 5:**
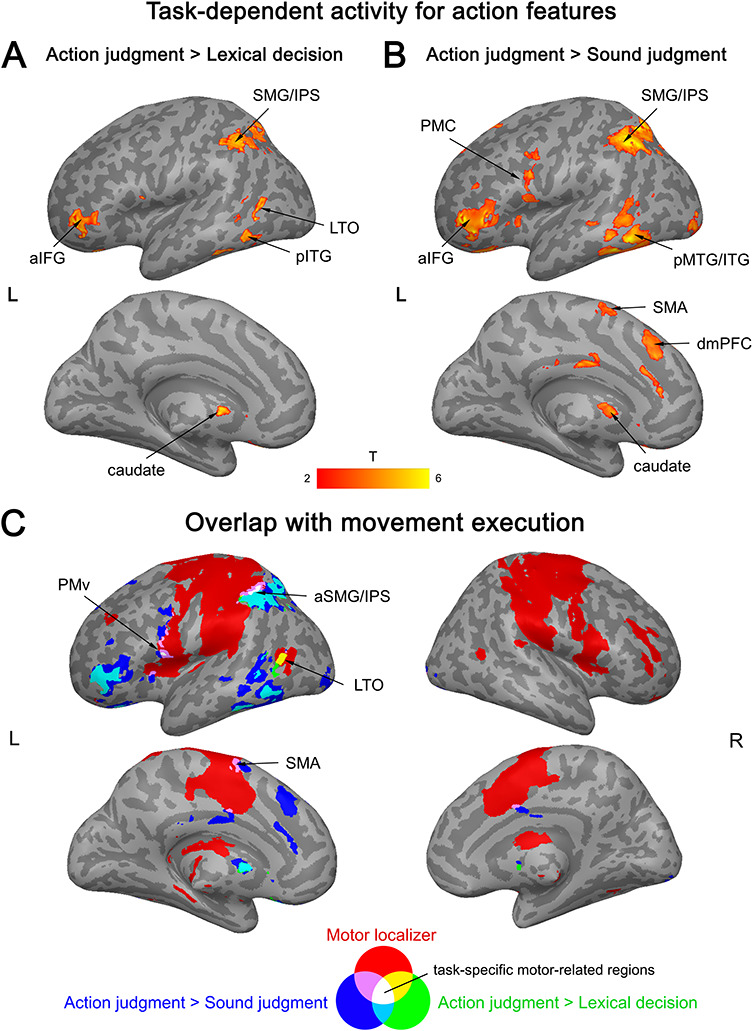
Interaction between activation for action features (high > low action words) and task. (*A*) Stronger activation during action judgments than lexical decisions. (*B*) Stronger activation during action judgments than sound judgments. (*C*) Activation overlap between the interaction and movement execution. All activation maps were thresholded at *q* < 0.05 FDR-corrected (extent > 20 voxels).

Several regions showed significantly stronger activation for high as compared to low action words in the action judgment task than in both other tasks (as determined by the conjunction of the TASK × ACTION interactions). These areas included left aIFG, SMG/IPS (extending into SPL), pITG (extending into FG), caudate nucleus, ventral PMC (PMv), and right cerebellum (Fig. 5; Supplementary Table S10). Additionally, left SMA, dmPFC, and bilateral cingulate cortex were more active during action judgments as compared to sound judgments (Supplementary Table S8). Finally, left pMTG/LTO showed stronger activation during action judgments than lexical decisions (Supplementary Table S9).

Among these regions, left PMv, anterior SMG/IPS, pMTG/LTO, SMA, and right cerebellum overlapped with the motor localizer (Fig. 5*C*). In contrast, no overlap was found in left aIFG, pITG/FG, dmPFC, caudate nucleus, more anterior parts of pMTG, and more posterior parts of SMG/IPS.

#### Sound Features

**Figure 6:**
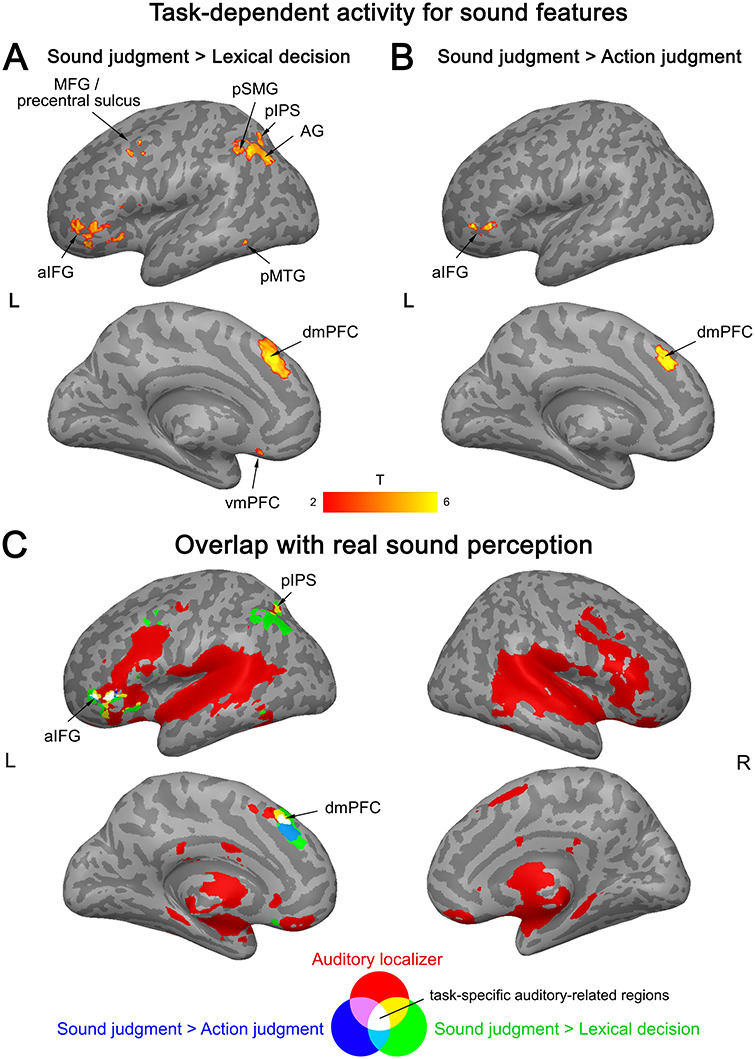
Interaction between activation for sound features (high > low sound words) and task. (*A*) Stronger activation during sound judgments than lexical decisions. (*B*) Stronger activation during sound judgments than action judgments. (*C*) Activation overlap between the interaction and real sound perception. All activation maps were thresholded at *q* < 0.05 FDR-corrected (extent > 20 voxels).

The strongest evidence for task-dependent activation for sound features of concepts was found in left aIFG and dmPFC. These regions showed significantly stronger activation for high versus low sound words in the sound judgment task than in both other tasks (Fig. 6; Supplementary Table S13). Moreover, the left pIPL (including AG, pSMG, pIPS), MFG/PreCS, vmPFC, and pMTG were more strongly engaged for high versus low sound words in the sound judgment task relative to the lexical decision task (Supplementary Table S12).

Clusters in left pIPS, aIFG, and dmPFC overlapped with real sound perception, whereas clusters in left AG, pSMG, MFG/PreCS, and pMTG did not (Fig. 6*C*).

### Multimodal Conceptual Regions

**Figure 7:**
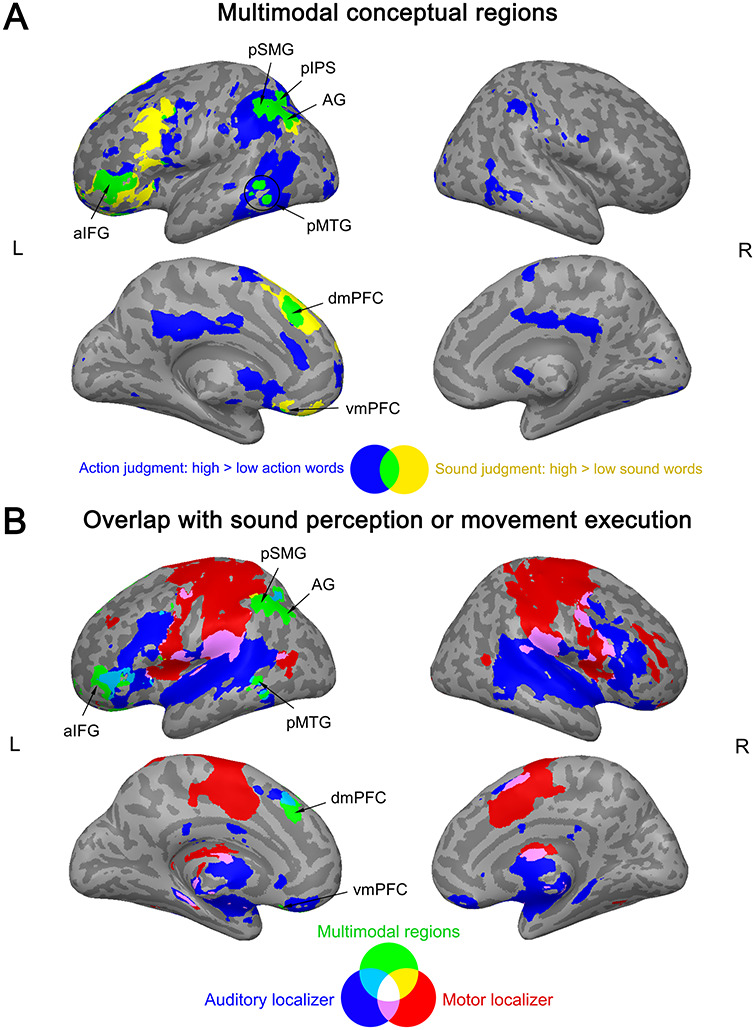
(*A*) Multimodal conceptual regions. Activation overlap (green) between the explicit retrieval of action features (blue) and sound features (yellow). (*B*) Overlap between multimodal regions (green) and the auditory localizer (blue) or motor localizer (red). All activation maps were thresholded at *q* < 0.05 FDR-corrected (extent > 20 voxels).

Finally, we tested for regions that were commonly engaged during the explicit retrieval of action features (high > low action words during action judgments) and sound features (high > low sound words during sound judgments). Such multimodal activation was found in left posterior IPL (AG, pSMG, IPS), pMTG, aIFG, dmPFC, vmPFC, and right cerebellum (crus I/II) (Fig. 7*A*; Supplementary Table S14).

These regions were located largely *outside* auditory and motor systems (Fig. 7*B*): Left AG, pSMG, vmPFC, anterior-most IFG, as well as parts of left pMTG, dmPFC, and right cerebellum overlapped with neither the auditory nor motor localizer. A small cluster in left anterior IPS (aIPS) overlapped with the motor localizer, while overlap with the auditory localizer was found in left pIPS, more posterior parts of IFG (extending into insula), and portions of pMTG, dmPFC, and right cerebellum.

### Subject-Specific fROI Analyses

To characterize the complete response profiles of motor, auditory, and multimodal regions involved in conceptual processing, we performed a subject-specific fROI analysis. In contrast to standard group analyses that aggregate responses from the same location in standard space across subjects, fROI analyses aggregate responses from the same *functional* region across subjects, resulting in higher sensitivity and functional resolution (i.e., the ability to separate adjacent but functionally distinct regions) (Fedorenko and Kanwisher 2009, 2011; Nieto-Castañón and Fedorenko 2012). fROI analyses are thus complementary to our whole-brain analyses: They allow us to determine whether regions defined functionally in individual subjects are indeed specific to action or sound features, or multimodal, and to what extent their feature-related activity is task-dependent. Different data of each subject were used for fROI definition and response estimation.

**Figure 8:**
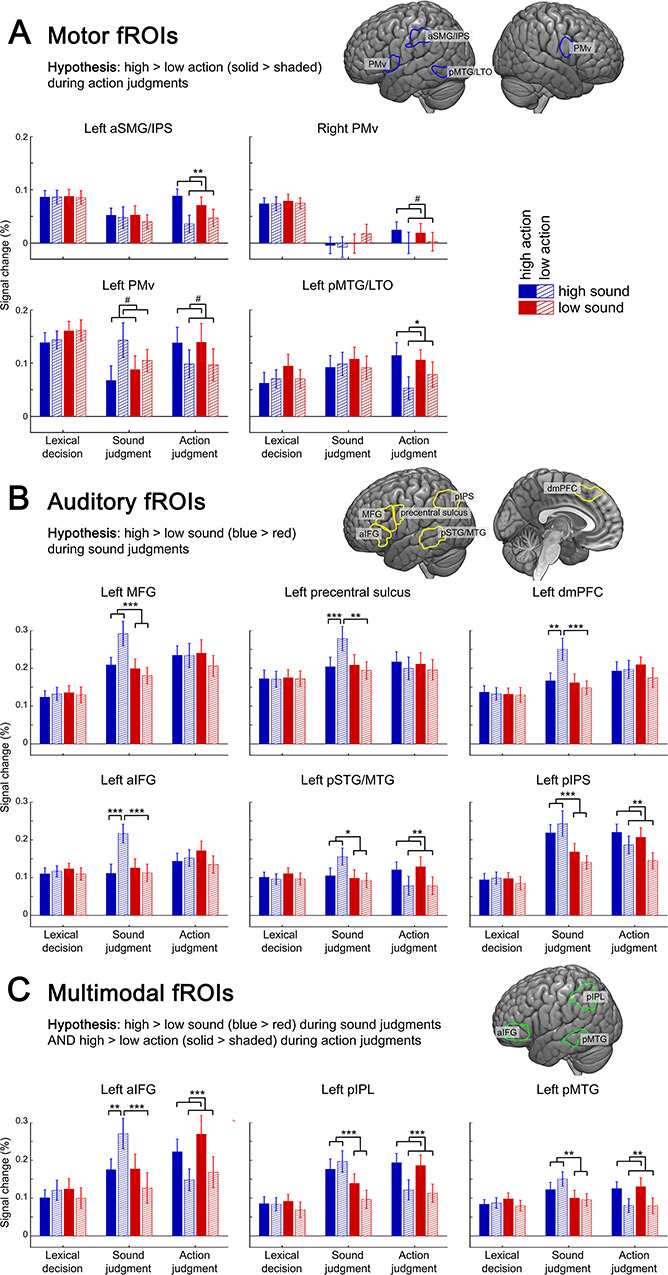
Response profiles for (*A*) motor fROIs, subject-specific regions engaged for action feature retrieval and the motor localizer; (*B*) auditory fROIs, subject-specific regions engaged for sound feature retrieval and the auditory localizer; and (*C*) multimodal fROIs, subject-specific regions engaged for both sound and action feature retrieval. Mean signal change (in %) is shown for each experimental condition; error bars represent standard error of the mean. ^*^P < 0.05, ^**^P < 0.01, ^***^P < 0.001, ^#^*P* < 0.1/did not survive multiple comparisons correction.

We identified motor fROIs (subject-specific regions engaged for action feature retrieval and the motor localizer) in left aSMG/IPS, pMTG/LTO, and left and right PMv (Fig. 8*A*); auditory fROIs (subject-specific regions engaged for sound feature retrieval and the auditory localizer) in left aIFG, MFG, PreCS, pIPS, pSTG/MTG, and dmPFC (Fig. 8*B*); and multimodal fROIs (subject-specific regions engaged for both sound and action feature retrieval) in left aIFG, pIPL, and pMTG (Fig. 8*C*).

A repeated-measures ANOVA revealed a REGION × TASK × SOUND × ACTION interaction (*F*(32,543) = 2.074, *P* = 0.01). We resolved this interaction using step-down ANOVAs within each fROI.

#### Motor fROIs

Motor fROIs in left aSMG/IPS and pMTG/LTO showed significant TASK × ACTION interactions, which were driven by a high > low ACTION effect during action judgments but not the other tasks (Fig. 8*A*; Supplementary Table S15 for statistics). Right PMv exhibited a similar, albeit nonsignificant, response pattern. Left PMv also showed a significant TASK × ACTION interaction, but this was driven by trends toward a high > low ACTION effect during action judgments and a low > high ACTION effect during sound judgments, potentially reflecting suppression of action-related activity when action features are irrelevant. Direct between-task comparisons revealed that all motor fROIs showed stronger activity for action features (high > low ACTION) during action judgments than during sound judgments and/or lexical decisions.

These results indicate that left aSMG/IPS, pMTG/LTO, and bilateral PMv are specific to action features in a task-dependent fashion, responding selectively to action features (not sound features) exclusively during action judgments.

#### Auditory fROIs

Auditory fROIs in left aIFG, PreCS, and dmPFC all showed TASK × SOUND × ACTION interactions, driven by a SOUND × ACTION interaction during sound judgments and no effects during the other tasks (Fig. 8*B*; Supplementary Table S16 for statistics). This interaction occurred as high sound–low action words produced stronger activity than the other conditions (which did not differ between each other). Left MFG showed a TASK × SOUND interaction, driven by a high > low SOUND effect during sound judgments but not during the other tasks. All of these regions were more strongly engaged for sound features (high > low SOUND) during sound judgments than during action judgments and/or lexical decisions.

These results indicate that auditory-related areas within left aIFG, PreCS, dmPFC, and MFG are specific to sound features in a task-dependent manner, responding to high (vs. low) sound words (MFG) or even only to high sound–low action words (aIFG, PreCS, dmPFC) selectively during sound judgments.

Left pIPS and pSTG/MTG showed a distinct response profile: Both regions exhibited significant TASK × SOUND and TASK × ACTION interactions, which were driven by a high > low SOUND effect during sound judgments, a high > low ACTION effect during action judgments, and no effects during lexical decisions. Activity for sound features (high > low SOUND) was significantly stronger during sound judgments than action judgments or lexical decisions, and activity for action features (high > low ACTION) was higher during action judgments than sound judgments or lexical decisions. This suggests that these areas are not specific to sound features but indeed multimodal, responding to both sound and action features when they are task-relevant, respectively (see below).

#### Multimodal fROIs

Both left pIPL and pMTG showed significant TASK × SOUND and TASK × ACTION interactions, which were driven by a high > low SOUND effect during sound judgments, a high > low ACTION effect during action judgments, and no effects during lexical decisions (Fig. 8*C*; Supplementary Table S17 for statistics). Left aIFG showed a slightly different response profile with a TASK × SOUND × ACTION interaction, driven by a SOUND × ACTION interaction during sound judgments which occurred as high sound–low action words produced stronger activity than all other conditions (which did not significantly differ). Like pIPL and pMTG, left aIFG showed a high > low ACTION effect during action judgments and no effects during lexical decisions. All three regions showed significantly stronger activation for sound features (high > low SOUND) during sound judgments than during both action judgments and lexical decisions and stronger activation for action features (high > low ACTION) during action judgments than during sound judgments and lexical decisions.

These findings provide strong evidence that left aIFG, pIPL, and pMTG contain multimodal and task-dependent areas involved in conceptual processing in individual subjects, responding to both action and sound features selectively when these are task-relevant.

## Discussion

In this study, we investigated the neural correlates of conceptual processing and their modulation by task. We found neural activation for action and sound features of concepts selectively when they were task-relevant in motor- and auditory-related areas, respectively, as well as in higher-level, multimodal regions. Both modality-specific and multimodal regions showed significantly stronger activity for a certain feature when that feature was task-relevant. These results provide strong evidence that the retrieval of conceptual features and recruitment of modality-specific perceptual–motor areas depend on the task. As an unexpected, novel finding, not only modality-specific, but also multimodal areas exhibited a task-dependent response to perceptual-motor features of concepts.

### Action Feature Retrieval Involves Motor-Related and Multimodal Regions

Exclusively during action judgments, action features of concepts produced widespread activation, which partially overlapped with the motor localizer in bilateral PMC, SMA, somatosensory areas, aSMG/IPS, pMTG/LTO, and cerebellum.

These regions represent secondary or association regions of the motor system, which are not involved in movement per se, but support movement planning or preparation (for reviews, see van Elk et al. 2014; Hardwick et al. 2018). PMC is associated with actions in the environment (Rizzolatti et al. 1988; Davare et al. 2009), whereas SMA is linked to actions that require little monitoring of the environment, such as self-generated actions (Deecke and Kornhuber 1978; Halsband et al. 1994; Debaere et al. 2003). The cerebellum controls the timing, strength, and precision of movement (Haggard et al. 1995; Wolpert et al. 1998; Ohyama et al. 2003) and contains somatotopic motor representations in its anterior and posterior lobes (Buckner 2013). aSMG/IPS is involved in the visual–motor control of object-directed actions (Haaland et al. 2000; Turella and Lingnau 2014), and pMTG/LTO represents different types of hand actions during execution, observation, and imagery (Lewis 2006; Oosterhof et al. 2010). The subject-specific fROI analysis revealed that action-related areas in left aSMG/IPS, pMTG/LTO, and bilateral PMv indeed specifically respond to action features when these are task-relevant and never respond to sound features.

Several of these regions have previously been implicated in action-related conceptual processing. For instance, Fernandino et al. (2016a) found that the relevance of action features to word meaning correlated with activation during concreteness judgments in bilateral aSMG, pMTG/LTO, and somatosensory areas. A meta-analysis by Binder et al. (2009) showed that left pMTG/LTO and SMG were the only regions with consistent activation across neuroimaging studies for processing of words referring to manipulable artifacts as compared to living things and retrieval of action knowledge relative to other types of knowledge. Similarly, another meta-analysis found that only left pMTG/LTO, extending into SMG, was consistently activated across neuroimaging studies of action-related conceptual processing on words or pictures (Watson et al. 2013).

Overall, it seems that pMTG/LTO and SMG are more consistently engaged during action feature processing than PMC, SMA, somatosensory cortex, and cerebellum (Watson et al. 2013; van Elk et al. 2014). One intriguing hypothesis is that these latter areas, which arguably represent lower-level regions of the motor system, only come into play during tasks that require highly explicit or deep processing of action features, such as our action judgment task (cf. Watson et al. 2013). This notion is supported by a previous study that did *not* find activation for action features in PMC, SMA, or cerebellum during concreteness judgment (Fernandino et al. 2016a), a task that does not require the same extent of action feature processing as action judgment.

Note that our motor localizer was restricted to hand movements and therefore might not have engaged all brain regions involved in the complex object-directed actions associated with our high-action words. However, the localizer involved pinching and fist-making, which arguably resemble object-directed hand movements more closely than low-level motor tasks like finger tapping. Indeed, our motor localizer engaged pMTG/LTO and aSMG/IPS, two relatively high-level motor-related regions that are usually not engaged in finger tapping (Mostofsky et al. 2006; Gountouna et al. 2010).

Crucially, retrieval of action features also engaged regions *outside* motor-related areas. These included areas that have previously been proposed to constitute heteromodal regions involved in conceptual processing (left AG, pSMG, aIFG, and mPFC; Binder et al. 2009)—a crucial result we will return to below.

### Sound Feature Retrieval Involves Auditory-Related and Multimodal Regions

Sound features selectively elicited significant activation during sound judgments. This activation did not overlap with the perception of scrambled sounds; thus, we found no evidence for an involvement of early auditory cortex. However, activation overlapped with the perception of real object sounds in regions implicated in high-level auditory processing, including left IFG, MFG/PreCS, and pMTG. These areas respond more strongly to recognized environmental sounds than unrecognized time-reversed versions of the same sounds (Lewis et al. 2004). Moreover, left IFG and MFG are more strongly engaged during the recall of sounds than pictures (Wheeler et al. 2000). The homologue region in the monkey (left ventrolateral prefrontal cortex) contains neurons representing abstract sound categories (for a review, see Romanski and Averbeck 2009).

Notably, the subject-specific fROI analysis showed that the response of some auditory fROIs (left aIFG, PreCS, dmPFC) was mainly driven by sound judgments on high sound–low action words. This is striking since this condition was also associated with the lowest response accuracy, which might reflect that sound features of high sound–low action words are not associated with corresponding actions, making them more demanding to retrieve due to a lack of action–sound coupling (Lemaitre et al. 2018). One might wonder whether activity of these regions could solely reflect task demands, that is, domain-general executive control processes. Several arguments speak against this view. Firstly, we controlled for difficulty differences between trials and conditions by removing error trials from the analysis and including a response time regressor. Secondly, auditory fROIs were defined via overlap with the auditory localizer, in which subjects merely listened to sounds—a simple task that required little executive processing. The overlap instead suggests that activation reflected engagement of auditory representations. Thirdly, the implicated regions have previously been associated with both high-level auditory processing (Wheeler et al. 2000; Lewis et al. 2004; Romanski and Averbeck 2009) and sound-related conceptual processing (Kellenbach et al. 2001; Kiefer et al. 2008; Fernandino et al. 2016a)—results that cannot be accounted for by task difficulty alone. Finally, the response profile of auditory fROIs was not a mirror image of behavioral performance: Performance differences were also seen during action judgments (see Fig. 2), but auditory fROIs responded selectively during sound judgments. Auditory fROIs thus showed modality- and task-specificity for sound features.

However, it is possible that some of the regions engaged during sound feature retrieval support the controlled retrieval of sound feature representations, rather than sound feature representation per se. A region representing sound features would be expected to activate not only for high sound–*low* action words but also for high sound–*high* action words. A selective response when conceptual retrieval demands are high seems more consistent with a role in conceptual-semantic control than representation (Jefferies 2013; Noonan et al. 2013; Lambon Ralph et al. 2016). The fact that activation for sound feature retrieval overlapped with perception of real object sounds, but not scrambled sounds, is consistent with the engagement of more abstract conceptual processes (Simanova et al. 2014). This is corroborated by the fact that especially left aIFG (Thompson-Schill et al. 1997; Wagner et al. 2001) and sometimes dmPFC (Alexander et al. 1989; Binder and Desai 2011) have previously been implicated in the controlled retrieval and/or selection of conceptual representations.

Several previous studies have implicated left pMTG in sound feature processing. In a lexical decision task, Kiefer et al. (2008) found that words with a high versus low relevance of sound features activate a region in left pMTG that was also engaged during real sound perception and overlaps with the pMTG region activated for sound features in our study. This sound-related pMTG subregion can be dissociated from a more posterior subregion that is engaged for action-related, but not sound-related concepts (Kiefer et al. 2012b) and overlaps with the pMTG/LTO area activated for action features in our study. Finally, a patient with a focal lesion in left pSTG/MTG was selectively impaired at processing sound-related, but not non-sound-related concepts, suggesting that this area is indeed causally relevant for processing sound features of concepts (Trumpp et al. 2013a; see Bonner and Grossman 2012 for corroborating evidence). However, in our study, the entire left pMTG region activated during the retrieval of sound features was also engaged during the retrieval of action features. This converges with previous evidence that the same region in left pMTG is modulated by both sound features and visual–motion features of concepts (Fernandino et al. 2016a). Moreover, left pMTG previously showed greater activity for both sound and action verbs as compared to pseudowords in a lexical decision task (Popp et al. 2019b). This area might therefore represent a multimodal, rather than sound-specific, region involved in conceptual processing. Indeed, the subject-specific fROI analysis revealed that even the portion of pMTG that overlapped with the auditory localizer was engaged for both sound and action feature retrieval. A similar multimodal response profile was found in left pIPS.

Crucially, activation for sound feature retrieval extended *beyond* the auditory localizer to left AG, pSMG, aIFG, and mPFC. Note that these regions were also engaged during the explicit retrieval of action features and did not overlap with the motor localizer, suggesting that they represent high-level, multimodal regions involved in conceptual processing.

Overall, the observed overlap of modality-specific activity patterns for conceptual feature retrieval and core regions for auditory or motor processing supports grounded theories of conceptual processing, which assume the retrieval of conceptual knowledge to involve a partial reinstatement of activity in perceptual–motor areas during actual perception and action (Pulvermüller 1999; Barsalou 2008; Kiefer and Pulvermüller 2012). However, our results are inconsistent with the view that conceptual processing relies *exclusively* on modality-specific perceptual-motor regions (e.g., Allport 1985) because the retrieval of sound or action features also involved higher-level, multimodal areas.

### Recruitment of Multimodal Regions during Conceptual Processing

Notably, several regions were engaged both during the explicit retrieval of action features and sound features, including left posterior IPL (AG, pSMG, pIPS), pMTG (anterior to LTO), aIFG, mPFC, and right cerebellum. These areas largely did not overlap with either the motor or auditory localizers, suggesting that they support more abstract representations than perceptual–motor areas.

Except for the right cerebellum, all of these regions have previously been described as “heteromodal” regions involved in conceptual processing, that is, regions engaged in the processing of all concepts, irrespective of their perceptual–motor content (Bonner et al. 2013; Binder 2016; Fernandino et al. 2016a, 2016b). This is supported by a meta-analysis of functional neuroimaging studies which showed that, among other regions, left posterior IPL, MTG, aIFG, and mPFC consistently show stronger activation for meaningful as compared to meaningless stimuli (Binder et al. 2009). It has been proposed that posterior IPL and parts of MTG act as heteromodal “convergence zones” at the top of a hierarchy integrating modality-specific representations into increasingly abstract representations (Binder and Desai 2011; Price et al. 2015). In contrast, prefrontal areas—especially left aIFG—appear to support the controlled retrieval and/or selection of conceptual representations (Thompson-Schill et al. 1997; Wagner et al. 2001; Jefferies 2013; Hartwigsen et al. 2016).

Crucially, the fact that these regions were sensitive to both action and sound features individually suggests that their representations are not amodal (i.e., completely abstracted away from modality-specific perceptual-motor information), but multimodal, that is, they retain modality-specific information about the individual features they integrate (cf. Barsalou 2016; Binder 2016; Fernandino et al. 2016a, 2016b). Note that this does not preclude the additional contribution of amodal regions to conceptual processing. For instance, it has been proposed that the ATL constitutes such an amodal “hub” (Lambon Ralph et al. 2016). In line with this view, the ATL was activated for words versus pseudowords in the lexical decision task (see Supplementary Fig. S2), indicating that it contributes to conceptual processing in general (cf. Binder et al. 2009).

Surprisingly, the right cerebellum also emerged as one of the regions engaged during the retrieval of both action and sound features. While the cerebellum is not included in contemporary models of conceptual processing (e.g., Binder and Desai 2011; Lambon Ralph et al. 2016), increasing evidence suggests that it contributes to higher cognitive processes and not just to movement planning and execution (for reviews, see Strick et al. 2009; Buckner 2013). Indeed, the subregion of the cerebellum activated during action and sound feature retrieval in the present study shows selective resting-state functional connectivity with *all* other multimodal conceptual regions identified here, that is, posterior IPL, pMTG, aIFG, and mPFC (Krienen and Buckner 2009; Buckner et al. 2011). The fact that this area did not overlap with the motor localizer further strengthens the view that it constitutes a nonmotor, higher-level subregion of the cerebellum.

Notably, the subject-specific fROI analysis identified two distinct functional subregions within the left aIFG: a sound-specific and a multimodal subregion. This illustrates the advantages of subject-specific fROI analyses, which yield higher sensitivity and functional resolution (i.e., the ability to separate adjacent but functionally distinct areas) than standard group analyses (Fedorenko et al. 2010; Nieto-Castañón and Fedorenko 2012). Importantly, this analysis confirmed that multimodal conceptual regions (sets of voxels activated during both sound and action feature retrieval) exist in individual subjects.

**Figure 9:**
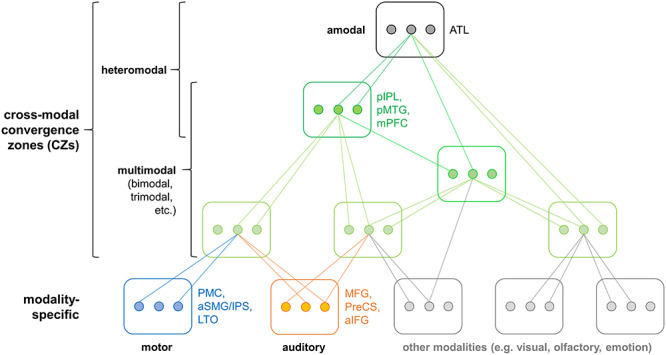
A new model of the neural architecture underlying conceptual representation. *Modality-specific* representations are integrated into increasingly abstract representations via multiple levels of cross-modal CZs. Heteromodal CZs, which receive input from all modalities, can be subdivided into *multimodal* regions that retain modality-specific information and *amodal* regions that do not. Boxes represent brain regions and dots represent individual representational units that converge onto a more abstract representation at a higher level. Note that this is merely a simplified schema and more hierarchy levels probably exist. The model is a synthesis of our current results and previous theories (Binder and Desai 2011; Lambon Ralph et al. 2016; Fernandino et al. 2016a).

Overall, our results support theories that assume conceptual processing to rely on both modality-specific perceptual–motor regions and cross-modal^1^ convergence zones (CZs), such as the “embodied abstraction” (Binder and Desai 2011; Fernandino et al. 2016a) and “hub-and-spokes” (Patterson et al. 2007; Lambon Ralph et al. 2016) models. Whereas the hub-and-spokes model singles out the ATL as the main “hub” for conceptual knowledge, the embodied abstraction view proposes a hierarchy of cross-modal CZs in the inferior parietal, temporal, and medial prefrontal cortices. Our results support the embodied abstraction view as we found evidence for multimodal conceptual processing in left pIPL, pMTG, and mPFC (among other regions). While recent versions of the hub-and-spokes view propose the ATL to be “graded,” with subregions closest to modality-specific cortices preferring the respective modalities (Lambon Ralph et al. 2016), we found no modality-specific effects in the ATL. However, as mentioned above, the ATL might constitute an amodal region supporting conceptual processing. Therefore, our results seem consistent with a theory that combines both the embodied abstraction and hub-and-spokes views (Fig. 9): Conceptual processing may rely on a representational hierarchy from modality-specific perceptual–motor regions to multiple levels of cross-modal CZs, including multimodal (bimodal, trimodal, etc.) up to heteromodal CZs, which receive input from all modalities (cf. Damasio 1989; Mesulam 1998; Simmons and Barsalou 2003; Binder and Desai 2011; Margulies et al. 2016). As a novel distinction, we subdivide heteromodal CZs into two classes: 1) Heteromodal CZs that are *multimodal* themselves, that is, retain modality-specific information and 2) *amodal* regions that completely abstract away from modality-specific input. Amodal regions thus occupy the top of the hierarchy with the highest level of abstraction. Together with previous evidence, our data suggest that high-level *multimodal* CZs include left pIPL, pMTG, and mPFC (Binder et al. 2009; Binder 2016), and the ATL functions as an *amodal* hub (Jefferies 2013; Lambon Ralph et al. 2016). In addition to the representational hierarchy, control regions (especially left aIFG) support the controlled retrieval and/or selection of conceptual representations (Thompson-Schill et al. 1997; Wagner et al. 2001; Noonan et al. 2013).

### Task Dependency of Conceptual Feature Retrieval

Many of the regions that were engaged during the retrieval of action or sound features were more strongly activated when the respective feature was task-relevant. These included both modality-specific perceptual-motor areas and multimodal regions.

Together with the finding that action or sound features only produced significant activity when they were explicitly required by the task, these results suggest that perceptual–motor features are selectively retrieved when they are task-relevant. Moreover, they support the view that the engagement of modality-specific perceptual–motor areas in conceptual processing strongly depends on the task (Hoenig et al. 2008; Binder and Desai 2011; Willems and Casasanto 2011; Kemmerer 2015; Yee and Thompson-Schill 2016). For example, Hoenig et al. (2008) found that visual- and motor-related areas showed stronger activity when a nondominant conceptual feature (i.e., visual for artifacts; action for natural items) than a dominant feature had to be verified for an object noun. Van Dam et al. (2012) found left IPL, pIPS, and pMTG to be more active for words with a high relevance of both action and color features during a task focusing on action than a task focusing on color. Another study reported that a color-sensitive region in left fusiform gyrus responded more strongly when the task required more detailed color knowledge (Hsu et al. 2011). Finally, Borghesani et al. (2019) showed that a similar set of brain regions as those engaged for action feature retrieval in our study (bilateral SMG/IPS, pMTG/LTO, and aIFG) exhibits higher activity when two object pictures are compared for movement than for typical location, regardless of object category (animals, tools, non-tool artifacts).

The results of these studies corroborate our findings of task-dependent engagement of modality-specific perceptual–motor regions for conceptual feature retrieval. In line with our conclusions, van Dam et al. infer from their results that motor-related regions show stronger activity for action-related concepts when action features are task-relevant. At first sight, Hoenig et al.’s and Borghesani et al.’s findings might seem at odds with our result of increased activity for high versus low action words during action judgments: Hoenig et al. found stronger activity for nondominant than dominant features, and Borghesani et al. failed to detect an interaction with stimulus category. However, this may merely reflect differences in the experimental design. In Hoenig et al.’s study, a feature had to be verified for a subsequently presented object concept on every trial, which seemed to require increased processing in modality-specific areas when the feature was nondominant. Similarly, Borghesani et al.’s movement task required retrieval of motion features for all stimuli. In contrast, our action judgment task exclusively required retrieval of action feature representations for high action words; low action words only necessitated confirmation that they lacked a (strong) action feature representation. Therefore, in all cases, increased activity in perceptual–motor regions seems to reflect increased activation of modality-specific conceptual features when they are task-relevant.

However, except for Hsu et al., none of these previous studies tested for activation overlap with perception and action. It was thus unclear whether the task-dependent regions were indeed located within perceptual–motor systems. This is especially crucial for regions like left IPL or pMTG where modality-specific and higher-level multimodal regions lie side by side (see Figs 7 and 8). Secondly, Hoenig et al. and Hsu et al. confounded their task manipulation with stimulus differences, rendering it ambiguous whether activation differences were due to different tasks, different stimuli, or both. Thirdly, none of the previous studies independently manipulated the relevance of multiple perceptual–motor features at the same time, preventing the investigation of modality-specificity. While van Dam et al. manipulated the relevance of both action and color features, activation was not compared directly, but only against abstract words. In Hoenig et al.’s study, manipulation of visual and action relevance was nonorthogonal and confounded with stimulus category.

We addressed these limitations by directly comparing neural activity during different tasks on the same stimuli, allowing us to unambiguously attribute activation differences to task, and not stimulus, differences. Moreover, we directly tested for activation overlap with perception and action, which enabled us to determine which of the task-dependent regions were located within perceptual–motor cortices. Finally, we independently manipulated the relevance of both action and sound features for a concept, which allowed us to test whether a brain region was specific to action or sound features, or multimodal.

Surprisingly, not only modality-specific areas but also higher-level, multimodal regions showed a task-dependent response to sound or action features. Modality-specific areas are selectively sensitive to the single feature they represent when it is task-relevant, while multimodal areas seem selectively sensitive to any of the multiple features they bind when these are task-relevant. These findings suggest that the task modulates not just which levels of the processing hierarchy are engaged. Rather, the task modulates activity for individual perceptual–motor features at several, possibly all, levels of the hierarchy.

It should be noted that some studies found modality-specific activations even during shallow tasks, that is, implicit (Pulvermüller et al. 2005; Kiefer et al. 2008, 2012b; Sim et al. 2015) or passive tasks (Hauk et al. 2004; Hauk and Pulvermüller 2004), or when the stimulus was unattended (Shtyrov et al. 2004; Pulvermüller and Shtyrov 2006) or not consciously perceived (Trumpp et al. 2013b, 2014). These findings seem to contradict the proposal that perceptual–motor features are selectively retrieved when task-relevant. However, such effects have largely been observed when the pertinent feature was central to the concept. For instance, action verbs (e.g., “lick,” “kick,” or “pick”) engaged the motor cortex during shallow tasks (Hauk et al. 2004; Hauk and Pulvermüller 2004; Tettamanti et al. 2005). As action knowledge is crucial to the meaning of action verbs, activation of motor regions might be required even for shallow comprehension of action verbs. These findings are thus consistent with the view that perceptual–motor features are only activated when relevant in the current context.

Importantly, the fact that perceptual–motor features are not always activated during conceptual tasks does *not* entail that they are not essential components of a concept or that modality-specific brain regions are not functionally relevant for conceptual processing (Kemmerer 2015; Barsalou 2016). Instead, it implies that we need to abandon models of conceptual processing that assume a rigid, task-independent architecture and move to models that allow for task-dependent flexibility of the retrieval of different conceptual features and engagement of the brain systems that represent them (Hoenig et al. 2008; Binder and Desai 2011; Kiefer and Pulvermüller 2012; Kemmerer 2015).

Some authors have argued that perceptual–motor activations during conceptual tasks may be epiphenomenal (e.g., reflect post-conceptual mental imagery) and not *causally* relevant for conceptual processing (Mahon and Caramazza 2008). This issue cannot be addressed with correlative neuroimaging methods but requires methods that allow for causal inferences such as lesion or noninvasive brain stimulation studies (Walsh and Cowey 2000; Hartwigsen et al. 2015). Causal evidence for the involvement of perceptual–motor areas in conceptual processing is currently scarce and equivocal (Willems and Casasanto 2011; Hauk and Tschentscher 2013; Papeo et al. 2013). Hence, an important avenue for future research will be to investigate whether, and crucially, under which circumstances perceptual–motor regions causally support conceptual processing.

## Conclusions

In conclusion, our results support theories that assume conceptual processing to rely on a flexible, multi-level architecture grounded in the perceptual–motor systems. Firstly, conceptual processing involves both modality-specific perceptual–motor areas and higher-level, multimodal regions. Secondly, the retrieval of a certain perceptual–motor feature and engagement of modality-specific areas are strongly task-dependent. Crucially, we show for the first time that not only modality-specific areas but also multimodal regions are sensitive to a certain conceptual feature exclusively when this feature is task-relevant. These findings indicate that the task modulates conceptual feature processing throughout the hierarchy of functional neural networks.

## Supplementary Material

Supplementary material can be found at: http://www.cercor.oxfordjournals.org/

## Funding

Max Planck Society; German Research Foundation (DFG, HA 6314/3–1, HA 6314/4–1 to G.H.).

## Notes

We thank Annika Tjuka for her tremendous help during data acquisition. We also thank Anke Kummer, Nicole Pampus, and Sylvie Neubert for acquiring participants and assisting the fMRI measurements. Moreover, we thank Toralf Mildner for implementing the dual-echo fMRI sequence and providing the code to combine the images, as well as Maren Grigutsch for programming the sound scrambling algorithm. We are also grateful to Marie Beaupain and Maike Herrmann for their assistance in stimulus creation and piloting. Finally, we thank four anonymous reviewers, Vincent Cheung, and Ulrich Kuhnke who contributed to a substantial improvement of this manuscript.

## Conflict of Interest

None declared.

## Supplementary Material

Supplementary_Material_bhaa010

## Footnotes

1 The term “cross-modal” denotes any region that integrates multiple modalities and thus subsumes multimodal and heteromodal areas.
