# Supplementary material for: Task-Dependent Recruitment of Modality-Specific and Multimodal Regions during Conceptual Processing
Source: Cereb Cortex. 2020 Mar 6;30(7):3938–59. doi: 10.1093/cercor/bhaa010 (PMC7264643; doi:10.1093/cercor/bhaa010)
Supplement: Supplementary_Material_bhaa010 [file supplementary_material_bhaa010.pdf]

Supplementary Material

Supplementary Materials and Methods

Table S1. Psycholinguistic measures for the four word categories (means; SD in parentheses).

	low sound	high sound	p	low action	high action	p
**Sound rating**	1.33 (0.29)	4.92 (0.62)	**$<10^{-113}$**	2.92 (1.67)	3.34 (2.03)	0.12
**Action rating**	3.19 (1.64)	3.4 (1.64)	0.37	1.73 (0.49)	4.86 (0.45)	**$<10^{-103}$**
**Visual rating**	4.21 (0.54)	4.07 (0.81)	0.15	4.16 (0.82)	4.13 (0.52)	0.77
**Familiarity rating**	5.52 (0.39)	5.47 (0.41)	0.41	5.45 (0.42)	5.55 (0.37)	0.08
**Letters**	6.23 (1.61)	6.36 (2.0)	0.61	6.27 (1.98)	6.32 (1.63)	0.84
**Syllables**	2.21 (0.77)	2.29 (0.71)	0.44	2.22 (0.71)	2.28 (0.76)	0.56
**Lemma freq.**	5.33 (6.33)	5.56 (10.35)	0.85	4.74 (6.34)	6.14 (10.3)	0.26
**Bigram freq.**	254220.81 (136859.15)	231922.15 (122883.01)	0.24	254413.24 (139511.49)	231729.73 (119826.78)	0.23
**Trigram freq.**	145830.18 (85313.42)	132754.69 (76696.95)	0.27	146068.82 (84690.3)	132516.05 (77342.95)	0.25
**Orthographic neighbors**	7.41 (6.7)	6.09 (5.86)	0.15	7.05 (6.83)	6.44 (5.77)	0.5

Ratings were obtained from a total of 163 subjects who did not participate in the fMRI experiment. All other psycholinguistic measures were extracted from the *dlexDB* database (Heister et al., 2011; http://dlexdb.de/). Lemma, bigram and trigram frequencies and number of orthographic neighbors are given per one million words.

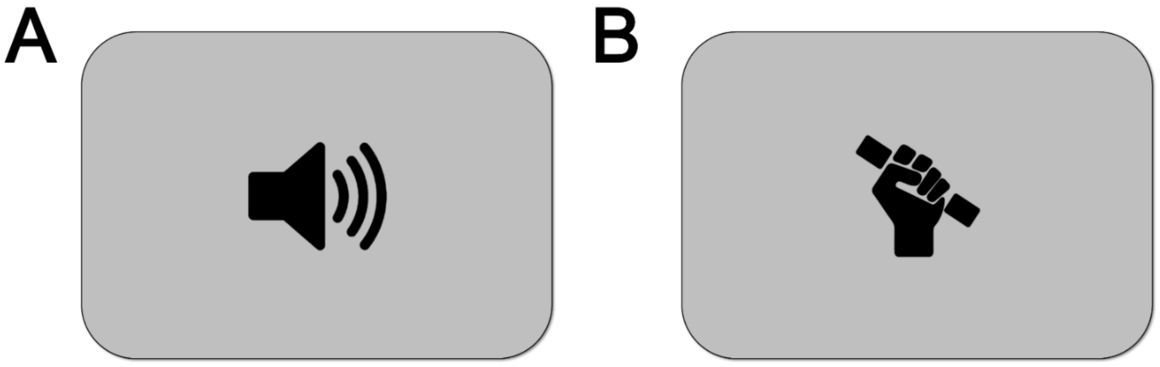

Figure S1. Cues presented at the beginning of mini-blocks for the sound judgment task (A) or action judgment task (B).

Supplementary Results

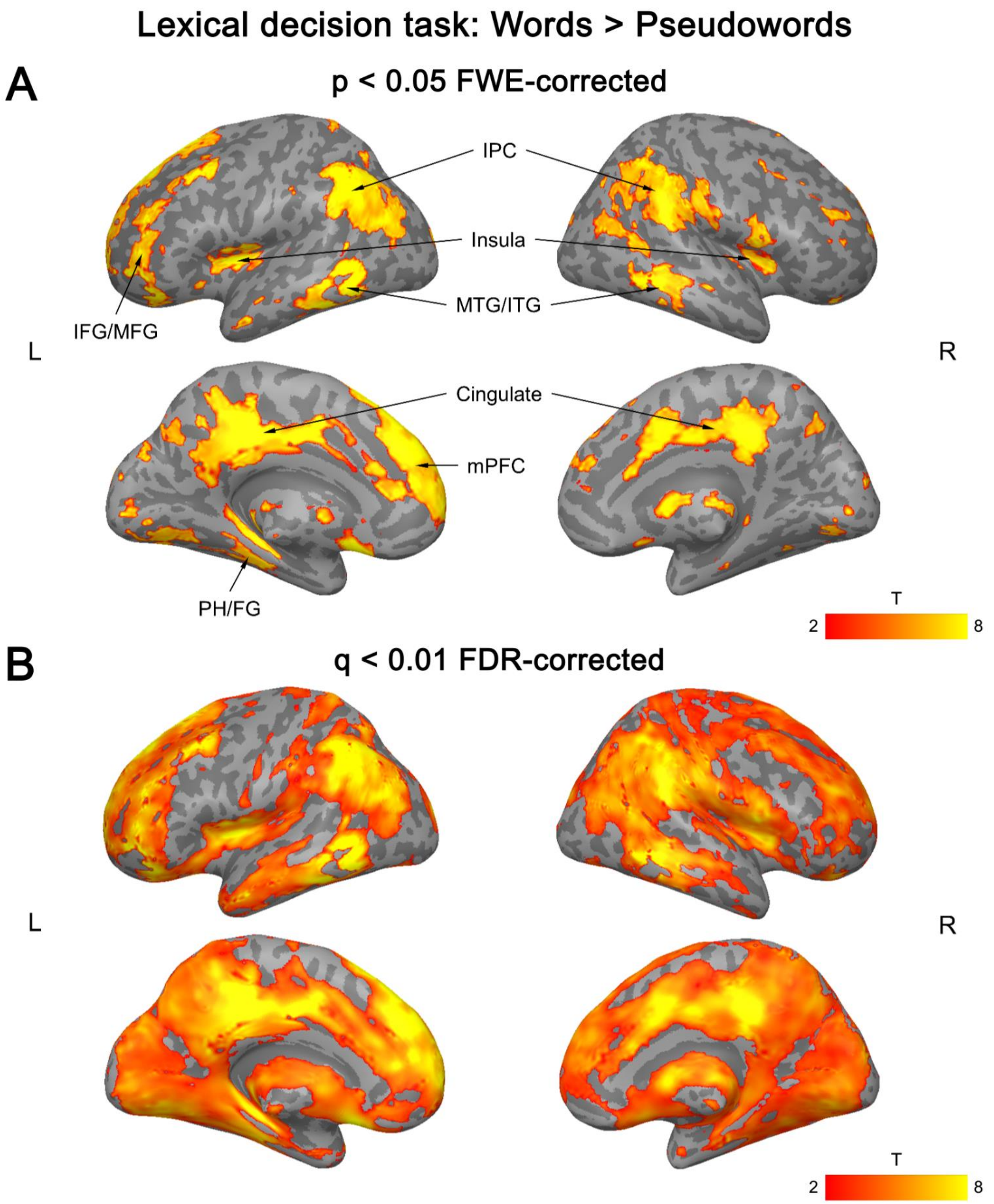

Figure S2. The “general” conceptual system (cf. Binder et al., 2009). Activation for words > pseudowords in the lexical decision task, at two different thresholds: (A) voxel-wise $p < 0.05$ family wise error (FWE) corrected; (B) voxel-wise $q < 0.01$ false discovery rate (FDR) corrected. FG = fusiform gyrus; IFG = inferior frontal gyrus; IPC = inferior parietal cortex; ITG = inferior temporal gyrus; MFG = middle frontal gyrus; MTG = middle temporal gyrus; mPFC = medial prefrontal cortex; PH = parahippocampal gyrus.

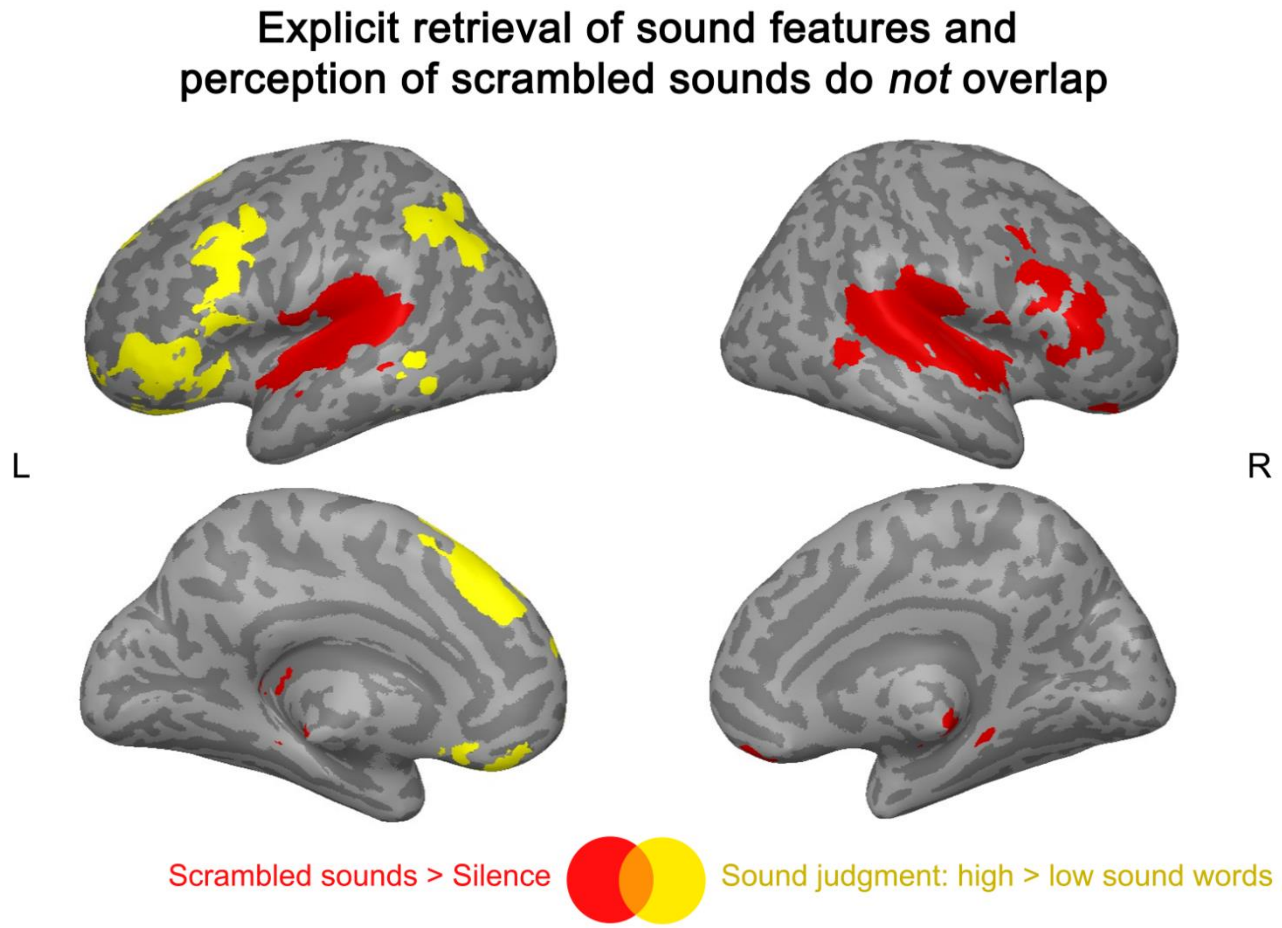

Figure S3. Activation for the explicit retrieval of sound features (yellow) does *not* overlap with activation for listening to scrambled sounds (red).

Activation Tables

The following tables show activations thresholded at q < 0.05 FDR-corrected (extent > 20 voxels). Up to 10 peaks per cluster are reported with T > 3.31 (p < 0.001 uncorrected) and more than 8 mm apart. Coordinates are in MNI space. Anatomical labels were determined using the SPM Anatomy toolbox (Version 2.2c; Eickhoff et al., 2005), the Harvard-Oxford atlas distributed with FSL (http://www.fmrib.ox.ac.uk/fsl/), and the human motor area template (http://lrnlab.org/; Mayka et al., 2006).

AG = angular gyrus; ACC = anterior cingulate cortex; MCC = middle cingulate cortex; PCC = posterior cingulate cortex; FG = fusiform gyrus; IFG = inferior frontal gyrus; IPL = inferior parietal lobule; IPS = inferior parietal sulcus; ITG = inferior temporal gyrus; LTO = lateral temporal-occipital junction; MTG = middle temporal gyrus; MFG = middle frontal gyrus; PFC = prefrontal cortex; dmPFC = dorsomedial PFC; vmPFC = ventromedial PFC; PMC = premotor cortex; PMd = dorsal PMC; PMv = ventral PMC; S1 = primary somatosensory cortex; S2 = secondary somatosensory cortex; SFG = superior frontal gyrus; SMA = supplementary motor area; SMG = supramarginal gyrus; SPL = superior parietal lobe; STG = superior temporal gyrus; a (prefix) = anterior; p (prefix) = posterior.

Table S2. Motor localizer: Hand movements > Rest.

Region	Cluster size (mm^3)	x	y	z	T
L/R M1, PMC, S1/S2, aSMG/IPS	234 719				
L M1 (4p)		-34	-24	55	21.53
R M1 (4p)		36	-22	55	19.87
L M1		-37	-17	65	19.53
L M1 (4p)		-32	-32	55	17.11
L SMA		-7	-7	52	15.99
L S1		-50	-20	55	15.31
R S1		50	-20	58	13.88
L S2 (OP1)		-44	-27	20	11.63
L parietal operculum		-47	-34	22	11.53
L thalamus (prefrontal)		-14	-20	8	11.19
L/R cerebellum	98 438				
L cerebellum (VI)		-20	-50	-22	18.53
R cerebellum (VI)		20	-50	-22	17.30
R cerebellum (VI)		26	-47	-28	16.82
R cerebellum (V)		6	-60	-12	16.00
R cerebellum (VIIIb)		16	-60	-52	14.84
L cerebellum (VIIIb)		-20	-57	-50	13.17
L cerebellum (V)		-4	-62	-18	12.16
R cerebellum (VIIIa)		8	-64	-32	11.99
L cerebellum (VIIIa)		-7	-64	-35	8.19
R frontal pole / MFG	6141				
R MFG		38	40	20	4.63
R frontal pole		33	46	-2	4.52
R frontal pole		26	43	-8	3.87
R frontal pole		23	46	-10	3.58
L pMTG/LTO	2500	-47	-62	8	7.23

L MFG	1469	-32	36	30	4.04
L MFG		-30	33	25	4.00
R pMTG/LTO	547	48	-60	5	4.07
R inferior occipital gyrus (V3v)	469	28	-94	-5	3.58
L superior orbital gyrus (Fo3)	453	-17	43	-15	3.81

Table S3. Auditory localizer.

**Region**	**Cluster size (mm^3)**	**x**	**y**	**z**	**T**
**Real sounds > Silence**					
L/R early auditory cortices, IFG	302 906				
L A1 (TE 1.0)		-50	-20	5	25.59
R A1 (TE 1.0)		56	-20	5	24.39
R STG (TE 3)		63	-30	10	21.39
R A1 (TE 1.0)		50	-10	0	19.68
R A1 (TE 1.1)		43	-22	8	19.68
R A1 (TE 1.2)		53	-2	-5	18.83
L STG (TE 3)		-67	-24	12	16.73
R STG (TE 3)		60	-10	-5	16.55
L A1 (TE 1.2)		-47	-10	-5	16.25
L STG		-54	-30	8	15.93
L/R dmPFC	5188				
R dmPFC (SFG)		6	6	62	5.67
L dmPFC (SFG)		-2	16	52	5.29
L dmPFC (SFG)		-2	28	48	4.54
L/R brainstem	1969				
R brainstem		6	-32	-38	4.88
R brainstem		18	-34	-38	3.98
L brainstem		-7	-34	-38	3.89
L IPS (hIP3)	1047	-30	-57	40	4.22
L IPS (hIP3)		-32	-64	48	3.64
L MCC	734	-2	3	25	4.95
**Scrambled sounds > Silence**					
R early auditory cortex	44 922				
R A1 (TE 1.1)		43	-22	10	24.32
R A1 (TE 1.0)		53	-20	5	22.67
R STG (TE 3)		63	-22	10	16.85
R STG/SMG (PF)		66	-30	12	16.67
R STG/SMG (PFcm)		46	-34	12	15.36
L early auditory cortex	31 531				
L A1 (TE 1.0)		-47	-22	8	23.73
L A1 (TE 1.1)		-40	-27	10	22.71
L STG		-47	-10	-5	13.26
L STG (TE 3)		-67	-27	12	10.32
L cerebellum	17 188				
L cerebellum (VII)		-12	-74	-40	7.86
L cerebellum (VIIb)		-24	-67	-50	6.42

L cerebellum (crus 1)		-10	-77	-30	5.65
L cerebellum (VIIb)		-32	-72	-55	4.99
L cerebellum (VI)		-22	-70	-28	4.73
L cerebellum (crus 1)		-34	-70	-28	4.31
R IFG	15 125				
R aIFG (pars triangularis)		46	36	2	5.84
R aIFG (pars triangularis)		50	33	20	5.28
R aIFG (pars triangularis)		43	26	18	5.11
R aIFG (pars triangularis)		53	26	22	4.94
R pIFG (pars opercularis)		40	8	30	4.19
L/R brainstem	5250				
R brainstem		16	-24	-10	5.85
R brainstem		3	-34	-8	5.63
L brainstem		-4	-32	-10	5.45
R cerebellum	2906				
R cerebellum (crus 2)		10	-80	-35	4.88
R cerebellum (crus 1)		16	-77	-25	4.29
R vmPFC	2703				
R vmPFC (Fo3)		28	33	-12	4.78
R vmPFC (Fp2)		8	53	-18	3.76
R brainstem	844	13	-37	-40	4.61
L brainstem	609	-10	-34	-40	4.54

Table S4. Action judgment task: high > low action words.

Region	Cluster size (mm^3)	x	y	z	T
L posterior temporal and inferior parietal cortices	63891				
L IPS (hIP2)		-47	-44	48	8.21
L SMG (PFm)		-44	-54	52	6.97
L pITG		-57	-42	-18	6.90
L SMG (PF)		-57	-37	45	6.51
L pMTG/LTO		-57	-54	2	6.08
L SPL (7A)		-37	-60	55	5.98
L pMTG/LTO		-64	-47	-2	5.65
L SMG (PF)		-60	-42	32	5.00
L IPS (hIP3)		-30	-64	42	4.63
L pITG		-62	-30	-20	4.52
R cerebellum	24078				
R cerebellum (crus II)		30	-77	-45	4.53
R cerebellum (crus I)		40	-62	-40	4.52
R cerebellum (crus II)		38	-70	-42	4.47
R cerebellum (lobule VI)		28	-60	-22	4.43
R cerebellum (lobule VI)		26	-70	-28	4.31
R cerebellum (lobule VIIb)		16	-72	-45	4.23
R cerebellum (crus I)		16	-80	-30	4.00
R cerebellum (crus I)		43	-60	-28	3.46
L anterior inferior frontal cortex, vmPFC	20203				
L caudate nucleus		-10	13	0	6.23
L aIFG (pars orbitalis)		-17	26	-20	6.09

L vmPFC (middle orbital gyrus)		-44	50	-2	5.56
L aIFG (pars orbitalis)		-44	43	-15	5.44
L aIFG (pars triangularis)		-47	46	8	4.37
L vmPFC (Fp1)		-17	63	-8	4.13
L vmPFC (Fp1)		-10	63	-15	4.08
L MFG		-44	46	18	4.02
L vmPFC (Fp1)		-10	66	5	3.67
L vmPFC (Fp1)		-30	60	-5	3.51
L dmPFC, SMA, ACC	17672				
L dmPFC (SFG)		-14	6	62	4.49
L dmPFC (SFG)		-4	33	32	4.30
L dmPFC (SFG)		-17	48	40	4.09
L ACC		-7	38	22	4.04
L dmPFC (SFG)		-20	13	65	4.00
L dmPFC (SFG)		-4	30	42	3.89
L ACC		-4	43	10	3.82
L dmPFC (SFG)		-17	33	55	3.81
L SMA		0	0	62	3.70
L dmPFC (SFG)		-12	3	75	3.65
L cingulate cortex	11719				
L PCC		-2	-32	32	5.34
L ACC		-4	-2	30	4.50
L premotor, somatosensory and inferior frontal cortex	11328				
L PMv		-47	3	22	4.48
L pIFG (pars opercularis)		-50	10	8	4.23
L insula		-34	18	-5	3.51
L insula		-30	16	5	3.51
L PMd		-50	6	45	3.44
L PMv		-50	6	35	3.32
L aIFG (pars triangularis)		-60	18	8	3.32
R inferior occipital cortex	5703				
R inferior occipital gyrus		23	-92	-5	3.72
R calcarine gyrus		16	-82	15	3.61
R posterior middle and inferior temporal gyri	5453				
R pMTG/LTO		66	-50	-5	4.12
R pMTG/LTO		48	-57	2	3.80
R pITG		63	-47	-12	3.56
R pITG		60	-57	-10	3.34
R SMG (PF)	4891	53	-37	52	4.36
L middle occipital gyrus	3844	-30	-90	-2	5.13
R basal ganglia	2297				
R pallidum		10	0	-5	5.53
R caudate nucleus		10	13	2	3.59
L cerebellum	2078				
L cerebellum (lobule VIII)		-12	-67	-50	3.50
L cerebellum (lobule VIIb)		-20	-77	-52	3.41
R premotor and somatosensory cortices	1125				

R PMv		63	6	22	3.52
R S1		66	-10	30	3.42
L thalamus	891	-10	-27	-10	3.96
L basal forebrain (Ch 4)	563	-12	-2	-12	3.74
R cerebellar vermis	453	6	-37	-20	3.54
R cerebellum (lobule VI)	453	33	-37	-32	3.44

Table S5. Conjunction: [Action judgment task: high > low action words] ∩ [Motor localizer: Hand movements > Rest].

Region	Cluster size (mm³)	x	y	z	T
L aSMG/IPS (extending into SPL), somatosensory cortex	11234				
L S2		-42	-42	50	5.73
L SPL (7PC)		-42	-44	55	5.65
L S2		-44	-40	48	5.42
L S2		-50	-40	58	5.22
L SMG (PFt)		-54	-32	45	4.96
L SMG (PF)		-57	-37	32	4.52
L SPL (7PC)		-34	-52	62	4.10
L SMG (PFop)		-50	-22	25	3.42
L PMC	6984				
L PMv		-47	3	20	4.39
L PMv		-50	10	8	4.23
L PMd		-50	3	45	3.40
L PMv		-50	6	35	3.32
R cerebellum	3922				
R cerebellum (lobule VI)		28	-60	-22	4.43
R cerebellum (lobule VI)		26	-67	-25	4.26
R cerebellum (crus I)		40	-60	-28	3.45
R cerebellum (crus I)		46	-57	-30	3.36
R aSMG/IPS, somatosensory cortex	2922				
R S1		50	-34	52	4.14
R S2		50	-37	58	3.94
R S2		40	-34	38	3.34
L/R (pre-)SMA	2719				
L SMA		0	0	62	3.70
L pre-SMA		-10	3	72	3.63
R SMA		3	0	70	3.48
L cerebellum (lobule VI)	2469	-20	-62	-15	3.84
L cerebellum (lobule VI)		-34	-64	-22	3.55
L cerebellum (lobule VI)		-27	-62	-22	3.43
R cerebellum (lobule VIIb)	2172	16	-72	-45	4.23
L pMTG/LTO	1984	-52	-62	5	5.52
L cerebellum (lobule VIIIa)	1406	-12	-67	-50	3.50
L MCC	922	-4	3	32	3.91
L thalamus	844	-10	-27	-10	3.96
R cerebellum (lobule VI)	453	33	-37	-32	3.44
R PMv	438	63	6	22	3.52
R inferior occipital gyrus (V3v)	438	28	-92	-5	3.44
R pMTG/LTO	391	46	-57	5	3.69
L insula	391	-30	16	5	3.51

Table S6. Sound judgment task: high > low sound words.

Region	Cluster size (mm^3)	x	y	z	T
L aIFG, MFG / precentral sulcus, vmPFC	28281				
L frontal pole		-47	46	-12	6.07
L vmPFC (Fo2)		-12	18	-18	5.57
L vmPFC (Fo3)		-20	33	-20	5.12
L MFG		-44	16	40	5.1
L aIFG (pars orbitalis)		-37	36	-18	5.07
L insula		-30	23	-10	4.87
L MFG		-40	10	48	4.69
L aIFG (pars triangularis)		-44	48	5	4.65
L aIFG (pars orbitalis)		-50	23	-10	4.3
L aIFG (pars triangularis)		-50	26	18	4.21
L posterior IPL (AG, pSMG, pIPS)	11547				
L AG (PGa)		-44	-62	50	6.14
L AG (PGa)		-37	-72	48	5.08
L IPS (hIP2)		-47	-44	50	4.87
L AG (PGp)		-50	-70	38	4.14
L dmPFC	11094				
L dmPFC (SFG)		-7	33	38	6.93
L dmPFC (SFG)		-4	30	48	5.7
L dmPFC (SFG)		-12	46	38	4.87
L dmPFC (SFG)		-10	28	58	4.39
L pMTG	1766	-57	-42	-12	4.83
R cerebellum (crus I)	1594	38	-77	-42	3.87
L SFG	594	-12	63	22	3.69

Table S7. Conjunction: [Sound judgment task: high > low sound words] ∩ [Auditory localizer: Real sounds > Silence].

Region	Cluster size (mm^3)	x	y	z	T
L aIFG, insula	6266				
L aIFG (pars triangularis)		-47	43	0	4.73
L aIFG (pars orbitalis)		-40	36	-18	4.60
L insula		-30	23	-5	4.19
L aIFG (pars orbitalis)		-50	20	-10	4.03
L aIFG, MFG / precentral sulcus	5391				
L aIFG (pars orbitalis)		-47	18	32	4.65
L aIFG (pars triangularis)		-50	26	18	4.21
L precentral sulcus		-37	3	38	3.34
L vmPFC	2156				
L vmPFC (Fo1)		-14	33	-20	4.09
L vmPFC (Fo1)		-7	50	-20	4.00
L vmPFC (Fo1)		-14	43	-18	3.55
L vmPFC (Fo1)		-10	33	-22	3.52
L vmPFC (Fo2)		-7	23	-20	3.48
L dmPFC (SFG)	1453	-2	28	48	4.54
L IPS (hIP3)	1047	-30	-57	40	4.22

L IPS (hIP3)		-32	-64	48	3.64
R cerebellum	516				
R cerebellum (crus I)		36	-70	-42	3.60
R cerebellum (crus II)		28	-74	-45	3.35
L pMTG	438	-62	-44	-12	3.93
L pMTG		-57	-47	-12	3.79

Table S8. Interaction: Action judgment task > Sound judgment task for high > low action words (inclusively masked with [Action judgment task: high > low action words]).

Region	Cluster size (mm^3)	x	y	z	T
L PMv, pIFG, insula	6172				
L pIFG (pars opercularis)		-40	8	25	5.90
L PMv		-37	3	35	5.25
L insula		-34	20	-5	5.07
L insula		-30	18	2	4.76
L pIFG (pars opercularis)		-52	16	-2	4.46
L pIFG (pars opercularis)		-60	18	10	4.03
L pIFG (pars opercularis)		-57	10	22	3.40
L dmPFC	5484				
L dmPFC (SFG)		-4	28	42	6.89
L ACC		-7	40	20	3.69
L dmPFC (SFG)		-10	43	42	3.64
L dmPFC (SFG)		-14	33	58	3.64
R lingual gyrus (V3v)	2375	23	-94	-8	5.57
L middle occipital gyrus (h0c4lp)	2328	-24	-94	-2	5.41
L (pre-)SMA	1391	-14	16	68	3.37
L caudate nucleus	1344	-10	16	0	4.53
L caudate nucleus		-10	3	8	3.51
L ACC	1016	-4	0	30	4.17
L thalamus	516	-12	-27	-10	4.38
L thalamus		-7	-17	-2	3.44
R pallidum	359	8	0	-5	3.60

Table S9. Interaction: Action judgment task > Lexical decision task for high > low action words (inclusively masked with [Action judgment task: high > low action words]).

Region	Cluster size (mm^3)	x	y	z	T
L SMG/IPS, SPL	8938				
L IPS (hIP2)		-44	-50	50	5.00
L SPL (7A)		-34	-62	55	4.25
L IPS (hIP3)		-30	-64	40	4.13
L pMTG/LTO, pITG	5172				
L pMTG		-52	-40	-10	4.78
L pMTG/LTO		-52	-60	5	4.23
L pITG		-57	-62	-8	3.99
L anterior inferior frontal cortex	2891				
L frontal pole		-42	50	-10	5.30

L MFG		-44	50	5	3.99
L aIFG (pars orbitalis)		-52	38	-10	3.86
R cerebellum	1547				
R cerebellum (crus I)		40	-60	-42	4.46
R cerebellum (crus I)		40	-62	-32	3.72
L caudate nucleus	828	-10	13	0	5.12
L aIFG (pars orbitalis)	719	-17	26	-20	5.12
R pallidum	594	10	0	-5	6.51
L PMv, pIFG	500				
L pIFG (pars opercularis)		-60	18	8	4.11
L pIFG (pars opercularis)		-50	13	0	3.44

Table S10. Conjunction of interactions: [Action judgment task > Sound judgment task for high > low action words] ∩ [Action judgment task > Lexical decision task for high > low action words] (both inclusively masked with [Action judgment task: high > low action words]).

**Region**	**Cluster size (mm^3)**	**x**	**y**	**z**	**T**
L SMG/IPS, SPL	8625				
L IPS (hIP2)		-44	-50	50	5.00
L SPL (7A)		-34	-62	55	4.25
L SPL (7A)		-30	-64	52	4.24
L IPS (hIP3)		-30	-64	40	4.13
L aSMG (PF)		-60	-40	45	3.49
L pMTG/ITG	3547				
L pMTG		-54	-42	-10	4.73
L pITG		-54	-60	-10	3.81
L anterior inferior frontal cortex	2875				
L frontal pole		-44	50	-8	4.99
L aIFG (pars orbitalis)		-40	46	-12	4.68
L aIFG (pars triangularis)		-44	48	5	3.89
L aIFG (pars orbitalis)		-52	38	-10	3.81
R cerebellum (crus I)	1125	40	-60	-40	4.11
R cerebellum (crus I)		43	-60	-30	3.24
L caudate nucleus	719	-10	16	0	4.53
L vmPFC	688				
L vmPFC (Fo3)		-20	26	-20	4.56
L vmPFC (Fo2)		-17	23	-18	4.27
L pIFG (pars opercularis)	500	-54	16	10	3.54
L pIFG (pars opercularis)		-52	13	8	3.54
L pIFG (pars opercularis)		-60	18	8	3.32
L pMTG/LTO	406	-62	-50	2	3.48

Table S11. Interaction: Sound judgment task > Action judgment task for high > low sound words (inclusively masked with [Sound judgment task: high > low sound words]).

Region	Cluster size (mm^3)	x	y	z	T
L dmPFC (SFG)	1406	-7	33	38	5.91
L aIFG (pars orbitalis)	891	-47	46	-8	5.61
L insula	94	-30	23	-10	4.58

Table S12. Interaction: Sound judgment task > Lexical decision task for high > low sound words (inclusively masked with [Sound judgment task: high > low sound words]).

Region	Cluster size (mm^3)	x	y	z	T
L posterior IPL (AG, pSMG, pIPS)	5953				
L AG (PGa)		-44	-62	50	6.04
L IPS (hIP2)		-50	-44	50	4.10
L dmPFC (SFG)	3281	-4	33	38	5.96
L anterior inferior frontal cortex	2719				
L frontal pole		-47	46	-12	4.98
L aIFG (pars orbitalis)		-37	38	-15	4.63
L frontal pole		-40	56	-5	3.94
L aIFG (pars orbitalis)		-54	33	-8	3.59
L aIFG (pars orbitalis)	734	-40	20	-10	4.28
L aIFG (pars orbitalis)		-30	26	-10	4.27
L MFG / precentral sulcus	672				
L MFG		-42	13	48	4.05
L precentral sulcus		-40	6	40	3.73
L vmPFC (Fo2)	406	-12	18	-18	4.77
L pMTG	328	-57	-40	-12	4.26
L aIFG (pars triangularis)	328	-52	18	8	3.84

Table S13. Conjunction of interactions: [Sound judgment task > Action judgment task for high > low sound words] ∩ [Sound judgment task > Lexical decision task for high > low sound words] (both inclusively masked with [Sound judgment task: high > low sound words]).

Region	Cluster size (mm^3)	x	y	z	T
L dmPFC (SFG)	1406	-7	33	38	5.91
L aIFG (pars orbitalis)	875	-47	46	-10	4.89

Table S14. Multimodal conceptual regions. Conjunction: [Action judgment task: high > low action words] ∩ [Sound judgment task: high > low sound words].

**Region**	**Cluster size (mm^3)**	**x**	**y**	**z**	**T**
L posterior IPL (AG, pSMG, pIPS)	10578				
L SMG (PFm)		-44	-60	50	5.88
L IPS (hIP2)		-47	-44	50	4.87
L IPS (hIP3)		-32	-64	42	4.58
L aIFG, vmPFC	8484				
L aIFG (pars orbitalis)		-44	43	-15	5.44
L vmPFC (middle orbital gyrus)		-47	48	-5	5.26
L vmPFC (Fo2)		-14	20	-20	4.76
L vmPFC (Fo3)		-20	30	-20	4.73
L dmPFC	3859				
L dmPFC (SFG)		-4	33	32	4.30
L dmPFC (SFG)		-4	30	42	3.89
L dmPFC (SFG)		-14	48	40	3.77
L ACC		-7	38	25	3.57
L dmPFC (SFG)		-14	33	52	3.40
L dmPFC (SFG)		-14	38	50	3.39
L pMTG	1766	-57	-42	-12	4.83
R cerebellum (crus I/II)	1594	38	-77	-42	3.87
L aIFG (pars triangularis)	672	-52	16	8	3.52

Subject-specific fROI Analysis

The following tables show the results of the subject-specific functional region of interest (fROI) analysis. A 4-way repeated-measures ANOVA with the factors REGION (all fROIs), TASK (lexical decision, sound judgment, action judgment), SOUND (high, low), and ACTION (high, low) revealed a significant 4-way interaction (F(32,543)=2.074, p=.01). We resolved this interaction using step-down ANOVAs within each fROI (post-hoc comparisons were Bonferroni-Holm corrected). Bold font highlights significant effects, italic font highlights trends (p < 0.1 / did not survive correction). SF = sound feature; AF = action feature; L = lexical decision; S = sound judgment; A = action judgment.

Table S15. Motor fROIs (identified using the conjunction [Action judgment: high > low action words] ∩ [Motor localizer: hand movements > rest]).

Region	Full ANOVA	Lexical decision	Sound judgment	Action judgment
Left aSMG/IPS	**TASK x ACTION (F(2,78)=3.886, p=.025)**	ACTION (F(1,39)=.046, p=.831)	ACTION (F(1,39)=.406, p=.528)	**ACTION (F(1,39)=11.070, p=.002)**<br>Task-specificity: **A vs. S for high > low AF (t(39)=2.228, p=.032); A vs. L for high > low AF (t(39)=2.595, p=.013)**
Left pMTG/LTO	**TASK x ACTION (F(2,78)=3.190, p=.049)**	ACTION (F(1,39)=.606, p=.441)	ACTION (F(1,39)=.092, p=.764)	**ACTION (F(1,39)=6.617, p=.014)**<br>Task-specificity: *A vs. S for high > low AF (t(39)=2.178, p=.035); A vs. L for high > low AF (t(39)=1.954, p=.058)*
Left PMv	**TASK x ACTION (F(2,78)=4.842, p=.024)**	ACTION (F(1,39)=.184, p=.670)	*ACTION (F(1,39)=5.629, p=.023)*	*ACTION (F(1,39)=3.191, p=.082)*<br>Task-specificity: **[A vs. S for high > low AF (t(39)=2.440, p=.019)];** [A vs. L for high > low AF (t(39)=1.565, p=.126)]
Right PMv	*TASK x ACTION (F(2,78)=2.498, p=.097)*	ACTION (F(1,39)=.784, p=.381)	ACTION (F(1,39)=.298, p=.588)	*ACTION (F(1,39)=3.593, p=.065)*<br>Task-specificity: *A vs. S for high > low AF (t(39)=2.102, p=.042);* A vs. L for high > low AF (t(39)=1.379, p=.176)

Table S16. Auditory fROIs (identified using the conjunction [Sound judgment: high > low sound words] ∩ [Auditory localizer: real sounds > silence]).

Region	Full ANOVA	Lexical decision	Sound judgment	Action judgment
Left aIFG	**TASK x SOUND x ACTION (F(2,78)=3.642, p=.032)**	SOUND (F(1,39)=.112, p=.740); ACTION (F(1,39)=.253, p=.618); SOUND x ACTION (F(1,39)=1.503, p=.228)	**SOUND x ACTION (F(1,39)=13.242, p<.001)**<br>**[high SF, low AF] vs. [high SF, high AF] (t(39)=3.934, p<.001); [high SF, low AF] vs. [low SF, low AF] (t(39)=4.184, p<.001);** [high SF, high AF] vs. [low SF, high AF] (t(39)=-.625, p=.536); [low SF, high AF] vs. [low SF, low AF] (t(39)=.613, p=.543)<br>Task-specificity: **S vs. A for high > low SF (t(39)=2.377, p=.022); S vs. L for high > low SF (t(39)=2.256, p=.03)**	SOUND (F(1,39)=.229, p=.635); ACTION (F(1,39)=.917, p=.344); SOUND x ACTION (F(1,39)=2.392, p=.13)
Left MFG	**TASK x SOUND (F(2,78)=6.750, p=0.003);** TASK x SOUND x ACTION (F(2,78)=2.332, p=0.105)	SOUND (F(1,39)=.204, p=.654)	**SOUND (F(1,39)=17.764, p<.001)**<br>Task-specificity: **S vs. A for high > low SF (t(39)=2.378, p=.025); S vs. L for high > low SF (t(39)=3.344, p=.002)**	SOUND (F(1,39)=.794, p=.378)
Left precentral sulcus	**TASK x SOUND x ACTION (F(2,78)=4.476, p=.020)**	SOUND (F(1,39)=.064, p=.802); ACTION (F(1,39)=.089, p=.767); SOUND x ACTION (F(1,39)=.014, p=.905)	**SOUND x ACTION (F(1,39)=11.002, p=.002)**<br>**[high SF, low AF] vs. [high SF, high AF] (t(39)=3.659, p<.001); [high SF, low AF] vs. [low SF, low AF] (t(39)=3.523, p=.001);** [high SF, high AF] vs. [low SF, high AF] (t(39)=-.220, p=.827); [low SF, high AF] vs. [low SF, low AF] (t(39)=.892, p=.378)<br>Task-specificity: *S vs. A for high > low SF (t(39)=1.720, p=.093); S vs. L for high > low SF (t(39)=2.322, p=.026)*	SOUND (F(1,39)=.249, p=.620); ACTION (F(1,39)=.821, p=.370); SOUND x ACTION (F(1,39)=.004, p=.952)
Left dmPFC	*TASK x SOUND x ACTION (F(2,78)=3.259, p=.053);* **TASK x SOUND (F(2,78)=7.224, p=.002); TASK x ACTION (F(2,78)=4.891, p=.01)**	SOUND (F(1,39)=.288, p=.595); ACTION (F(1,39)=.131, p=.719); SOUND x ACTION (F(1,39)=.051, p=.823)	**SOUND x ACTION (F(1,39)=6.988, p=.012)**<br>**[high SF, low AF] vs. [high SF, high AF] (t(39)=3.307, p=.002); [high SF, low AF] vs. [low SF, low AF] (t(39)=3.783, p<.001);**	SOUND (F(1,39)=.068, p=.796); ACTION (F(1,39)=.911, p=.346); SOUND x ACTION (F(1,39)=1.909, p=.175)

Region	Full ANOVA	Lexical decision	Sound judgment	Action judgment
			[high SF, high AF] vs. [low SF, high AF] (t(39)=.276, p=.791); [low SF, high AF] vs. [low SF, low AF] (t(39)=.712, p=.481)	
			Task-specificity:<br>**S vs. A for high > low SF (t(39)=2.909, p=.006); S vs. L for high > low SF (t(39)=3.250, p=.002)**	
Left pIPS	**TASK x SOUND (F(2,78)=9.098; p<.001);**<br>**TASK x ACTION (F(2,78)=5.443; p=.007)**	SOUND (F(1,39)=.395, p=.534);<br>ACTION (F(1,39)=.281, p=.599)	**SOUND (F(1,39)=23.250, p<.0001);**<br>ACTION (F(1,39)=.014, p=.908)	*SOUND (F(1,39)=5.326, p=.026);*<br>**ACTION (F(1,39)=11.157, p=.002)**
			Task-specificity:<br>**S vs. A for high > low SF (t(39)=2.558, p=.015); S vs. L for high > low SF (t(39)=4.227, p<.0001)**	Task-specificity:<br>**A vs. S for high > low AF (t(39)=2.719, p=.01); A vs. L for high > low AF (t(39)=2.733, p=.009)**
Left pSTG/MTG	**TASK x SOUND (F(2,78)=3.916; p=.025);**<br>**TASK x ACTION (F(2,78)=6.808; p=.002)**	SOUND (F(1,39)=.296, p=.589);<br>ACTION (F(1,39)=1.904, p=.175)	**SOUND (F(1,39)=6.536, p=.015);**<br>ACTION (F(1,39)=1.768, p=.191)	SOUND (F(1,39)=.153, p=.698);<br>**ACTION (F(1,39)=8.991, p=.005)**
			[high SF, high AF] vs. [low SF, high AF] (t(39)=-.220, p=.827); [low SF, high AF] vs. [low SF, low AF] (t(39)=.892, p=.378)	Task-specificity:<br>**A vs. S for high > low AF (t(39)=3.939, p<.001);** *A vs. L for high > low AF (t(39)=1.999, p=.053)*
			Task-specificity:<br>**S vs. A for high > low SF (t(39)=2.466, p=.018); S vs. L for high > low SF (t(39)=2.280, p=.028)**	

Table S17. Multimodal fROIs (identified using the conjunction [Action judgment: high > low action words] ∩ [Sound judgment: high > low sound words]).

Region	Full ANOVA	Lexical decision	Sound judgment	Action judgment
Left aIFG	**TASK x SOUND x ACTION (F(2,78)=3.712, p=.034)**	SOUND (F(1,39)=.007, p=.933);<br>ACTION (F(1,39)=.044, p=.834);<br>*SOUND x ACTION (F(1,39)=3.254, p=.079)*	**SOUND x ACTION (F(1,39)=12.458, p=.001)**<br><br>**[high SF, low AF] vs. [high SF, high AF] (t(39)=3.491, p=.001); [high SF, low AF] vs. [low SF, low AF] (t(39)=4.365, p<.0001);** [high SF, high AF] vs. [low SF, high AF] (t(39)=-.082, p=.935); *[low SF, high AF] vs. [low SF, low AF] (t(39)=1.945, p=.059)*<br><br>Task-specificity:<br>**S vs. A for high > low SF (t(39)=3.605, p<.001); S vs. L for high > low SF (t(39)=3.271, p=.002)**	SOUND (F(1,39)=1.376, p=.248);<br>**ACTION (F(1,39)=18.876, p<.0001);**<br>SOUND x ACTION (F(1,39)=.521, p=.475)<br><br>Task-specificity:<br>**A vs. S for high > low AF (t(39)=4.046, p<.001); A vs. L for high > low AF (t(39)=3.537, p=.001)**
Left pIPL	**TASK x SOUND (F(2,78)=8.148; p=.001);<br>TASK x ACTION (F(2,78)=7.478; p=.002)**	SOUND (F(1,39)=.308, p=.582);<br>ACTION (F(1,39)=1.696, p=.2)	**SOUND (F(1,39)=21.818, p<.0001);**<br>ACTION (F(1,39)=.535, p=.469)<br><br>Task-specificity:<br>**S vs. A for high > low SF (t(39)=2.846, p=.007); S vs. L for high > low SF (t(39)=4.5, p<.0001)**	SOUND (F(1,39)=.370, p=.546);<br>**ACTION (F(1,39)=18.742, p<.001)**<br><br>Task-specificity:<br>**A vs. S for high > low AF (t(39)=3.132, p=.003); A vs. L for high > low AF (t(39)=3.069, p=.004)**
Left pMTG	**TASK x SOUND (F(2,78)=5.512; p=.006);<br>TASK x ACTION (F(2,78)=6.222; p=.003)**	SOUND (F(1,39)=.187, p=.668);<br>ACTION (F(1,39)=1.185, p=.283)	**SOUND (F(1,39)=11.425, p=.002);**<br>ACTION (F(1,39)=.636, p=.430)<br><br>Task-specificity:<br>**S vs. A for high > low SF (t(39)=2.730, p=.009); S vs. L for high > low SF (t(39)=3.072, p=.004)**	SOUND (F(1,39)=.035, p=.852);<br>**ACTION (F(1,39)=10.265, p=.003)**<br><br>Task-specificity:<br>**A vs. S for high > low AF (t(39)=3.411, p<.002); A vs. L for high > low AF (t(39)=2.434, p=.02)**

References

Binder, J.R., Desai, R.H., Graves, W.W., Conant, L.L., 2009. Where Is the Semantic System? A Critical Review and Meta-Analysis of 120 Functional Neuroimaging Studies. Cereb. Cortex 19, 2767–2796.

Eickhoff, S.B., Stephan, K.E., Mohlberg, H., Grefkes, C., Fink, G.R., Amunts, K., Zilles, K., 2005. A new SPM toolbox for combining probabilistic cytoarchitectonic maps and functional imaging data. Neuroimage 25, 1325–1335.

Grinband, J., Wager, T.D., Lindquist, M., Ferrera, V.P., Hirsch, J., 2008. Detection of time-varying signals in event-related fMRI designs. Neuroimage 43, 509–520.

Heister, J., Würzner, K.-M., Bubenzer, J., Pohl, E., Hanneforth, T., Geyken, A., Kliegl, R., 2011. dlexDB – eine lexikalische Datenbank für die psychologische und linguistische Forschung. Psychol. Rundschau 62, 10–20.

Mayka, M.A., Corcos, D.M., Leurgans, S.E., Vaillancourt, D.E., 2006. Three-dimensional locations and boundaries of motor and premotor cortices as defined by functional brain imaging: A meta-analysis. Neuroimage 31, 1453–1474.
